# Hyperphosphorylation amplifies UPF1 activity to resolve stalls in nonsense-mediated mRNA decay

**DOI:** 10.1038/ncomms12434

**Published:** 2016-08-11

**Authors:** Sébastien Durand, Tobias M. Franks, Jens Lykke-Andersen

**Affiliations:** 1 Division of Biological Sciences, University of California San Diego, 9500 Gilman Drive, La Jolla, California 92093, USA

## Abstract

Many gene expression factors contain repetitive phosphorylation sites for single kinases, but the functional significance is poorly understood. Here we present evidence for hyperphosphorylation as a mechanism allowing UPF1, the central factor in nonsense-mediated decay (NMD), to increasingly attract downstream machinery with time of residence on target mRNAs. Indeed, slowing NMD by inhibiting late-acting factors triggers UPF1 hyperphosphorylation, which in turn enhances affinity for factors linking UPF1 to decay machinery. Mutational analyses reveal multiple phosphorylation sites contributing to different extents to UPF1 activity with no single site being essential. Moreover, the ability of UPF1 to undergo hyperphosphorylation becomes increasingly important for NMD when downstream factors are depleted. This hyperphosphorylation-dependent feedback mechanism may serve as a molecular clock ensuring timely degradation of target mRNAs while preventing degradation of non-targets, which, given the prevalence of repetitive phosphorylation among central gene regulatory factors, may represent an important general principle in gene expression.

## Abstract (web summary)

Gene expression is regulated by a range of mechanisms, including post-translational modifications such as phosphorylation. Here the authors present evidence for a feedback mechanism whereby hyperphosphorylation of UPF1 in response to delays in nonsense-mediated decay enhances recruitment of mRNA decay machinery.

## Graphical abstract

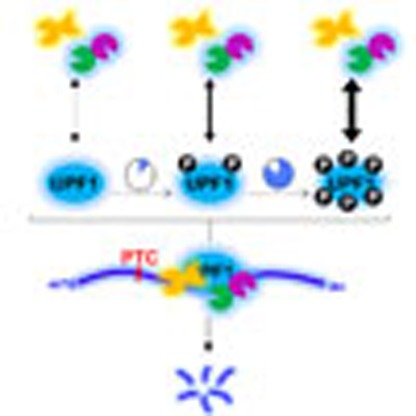

The correct control of gene expression requires coordination of multiple transcriptional and post-transcriptional processes. Thousands of genes or gene products use a shared pool of core gene expression machineries to carry out each step of gene expression. This is orchestrated by regulatory DNA- and RNA-binding factors, many of which target subsets of genes or gene products for regulation of specific steps in gene expression. However, the mechanisms by which gene-specific factors ensure timely regulation of their target genes or gene products in the face of changing demands for the core gene expression machineries is poorly understood.

RNA quality-control pathways maintain fidelity in gene expression by targeting faulty RNAs for decay^1^. Nonsense-mediated decay (NMD) is a quality-control pathway that monitors the integrity of gene expression by degrading messenger RNAs (mRNAs) that have acquired premature termination codons (PTCs), for example, through mutations, or errors in transcription or mRNA processing^2^,^3^,^4^,^5^,^6^. Given the potential for mRNAs with PTCs to cause accumulation of detrimental truncated protein products, the ability of NMD to degrade these mRNAs likely needs to be continuously sustained to avoid deleterious consequences, no matter the current availability of RNA decay machinery. Moreover, a critical aspect of NMD is that non-target mRNAs must remain immune to the pathway.

The detection of mRNAs with PTCs occurs during translation termination and is directed by the superfamily 1 RNA helicase UPF1 and co-factors^7^,^8^,^9^,^10^,^11^. In metazoans, subsequent to PTC recognition, UPF1 is phosphorylated by the phosphatidylinositol-kinase related kinase (PIKK) SMG1 at [S/T]Q motifs^12^,^13^. This activates downstream steps in the pathway carried out by the endonuclease SMG6 as well as the adaptor proteins SMG5, SMG7 and PNRC2, which connect UPF1 to the general decapping, deadenylation and exonucleolytic decay machineries^14^,^15^,^16^,^17^,^18^,^19^,^20^,^21^,^22^,^23^. While UPF1 specifically targets NMD substrates for degradation, our recent evidence suggests that UPF1 transiently associates with all translated mRNAs, but a mechanism dependent on UPF1 ATPase activity prevents the stable assembly of UPF1 with non-targets^24^.

Intriguingly, an evolutionary conserved property of UPF1 is its ability to undergo hyperphosphorylation^13^,^19^,^22^,^25^,^26^,^27^,^28^, a feature that is shared with many prominent factors in gene expression, including RNA polymerase II and SR proteins for which the importance of phosphorylation in gene expression is well described^29^,^30^,^31^. Metazoan UPF1 proteins contain a multitude of [S/T]Q motifs in the N- and C-terminal regions, the majority of which are evolutionarily conserved (for example, 19 in humans; Supplementary Fig. 1a). Specific [S/T]Q motifs in human UPF1 have been characterized as phosphorylation-dependent binding sites for downstream factors in the NMD pathway^10^,^17^,^32^,^33^, but the functional role of other [S/T]Q motifs and the significance of UPF1 undergoing hyperphosphorylation has remained uncharacterized.

Previous studies conducted to understand principles of UPF1 phosphorylation observed that phosphorylation of UPF1 increases on depletion of SMG5, SMG6 or SMG7 in *Caenorhabditis elegans* and human cells^10^,^22^,^25^,^28^. Those observations, together with an observed association of phosphatase 2A with SMG5-7 (refs 22, 25, 34), led to the conclusion that SMG5-7 promote UPF1 dephosphorylation. Here given the more recently demonstrated role of SMG5-7 in linking UPF1 to mRNA decay^14^,^16^,^17^,^18^,^19^,^21^,^23^, we considered the alternative but not necessarily mutually exclusive possibility that the increase in UPF1 phosphorylation on SMG5-7-depletion is caused by continuous phosphorylation of UPF1 as a consequence of a stall in the NMD pathway. Indeed, we find that multiple interventions that impair the NMD pathway downstream of UPF1 mRNA assembly, including mutation of the UPF1 ATPase and depletion of NMD-specific and general mRNA decay factors, all result in increased UPF1 phosphorylation. Moreover, UPF1 undergoes increased phosphorylation upon stimulation of the AU-rich element (ARE)-mediated mRNA decay pathway, which uses shared mRNA decay machinery. Mutational analyses demonstrate that no single phosphorylation site in UPF1 is essential for NMD, but multiple phosphorylation sites contribute to NMD efficiency with some sites being more important than others. Depletion of downstream NMD factors, SMG5 or SMG7, causes increased dependence of NMD on UPF1 hyperphosphorylation. Taken together, our observations suggest that UPF1 hyperphosphorylation serves as a feedback mechanism to ensure efficient degradation of mRNAs that stably assemble with UPF1. This could serve to ensure that NMD non-targets evade NMD despite their transient association with UPF1 (ref. 24), and to resolve stalls in the NMD pathway ensuring that mRNAs targeted for NMD are efficiently degraded even under conditions where downstream NMD-specific or general mRNA decay factors are limiting.

## Results

### Inhibiting late NMD steps increases UPF1 phosphorylation

**Figure 1:**
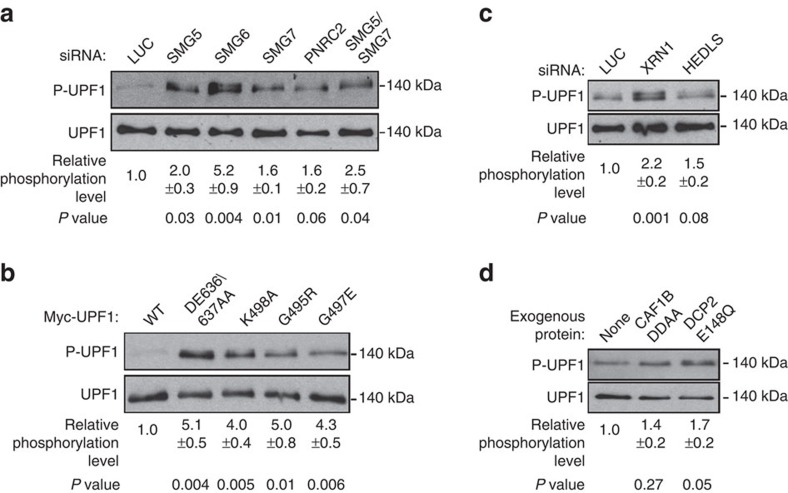
Inhibition of UPF1 ATPase and disruption of decay activities increase UPF1 phosphorylation. (**a**) Western blots monitoring phosphorylation levels of endogenous UPF1 in HeLa tet-off cells transfected with indicated siRNAs followed by IP for UPF1 and anti-phospho-[S/T]Q (P-UPF1) and anti-UPF1 (UPF1) western blotting. Indicated phosphorylation levels were calculated from at least three independent experiments by dividing the *P*-UPF1 signal with that from anti-UPF1 (UPF1) and are shown normalized to control (LUC) conditions±s.e.m. *P* values are indicated below panels and are relative to control conditions (paired two-tailed Student's *t*-test). (**b**) Same as in **a** in HeLa tet-off cells transfected with myc-tagged UPF1 wild-type (‘WT'), ATP-hydrolysis mutant (DE636/637AA) or ATP-binding mutants (K498A, G495R and G497E). Indicated phosphorylation levels were calculated as in **a** and are shown normalized to control (‘WT') conditions±s.e.m. (**c**) Same as in **a** in HeLa tet-off cells transfected with indicated siRNAs. UPF1 phosphorylation levels were quantified as in **a**. (**d**) Same as in **a** in HeLa tet-off cells transfected with plasmids coding for myc-tagged UPF1 together with an empty vector (none) or vectors expressing CAF1B DDAA or DCP2 E148Q. UPF1 phosphorylation levels were quantified as in **a** and are shown normalized to control (‘none') conditions±s.e.m.

To determine whether increased phosphorylation of UPF1 is a general response to stalls in downstream steps of the NMD pathway, we inhibited various steps of the NMD pathway and performed anti-UPF1 immunoprecipitation (IP) followed by western blotting using a general phospho-[S/T]Q antibody to monitor relative levels of [S/T]Q-phosphorylated UPF1. Consistent with the previous reports^10^,^22^,^25^,^28^,^35^, depletion of NMD factors SMG7, SMG6 or SMG5 resulted in increased UPF1 phosphorylation (Fig. 1a; depletion efficiencies shown in Supplementary Fig. 1b) and mutant UPF1 proteins with deficiencies in ATP hydrolysis (DE636/637AA) or ATP binding (K498A, G495R and G497E), known to become trapped on mRNAs and stalling NMD^24^,^36^,^37^, all accumulate with higher levels of phosphorylation than wild-type UPF1 (Fig. 1b). In addition, depletion of PNRC2 resulted in increased UPF1 phosphorylation (Fig. 1a, depletion efficiencies shown in Supplementary Fig. 1b,e). We also tested UPF1 phosphorylation levels following interventions that impair general mRNA decay factors. As seen in Fig. 1c,d, depletion of the 5′-to-3′ exonuclease XRN1 and the decapping activator HEDLS, as well as exogenous expression of a dominant negative form of the decapping enzyme DCP2 (DCP2-E148Q) augmented UPF1 phosphorylation (Fig 1c,d; see Supplementary Fig. 1c,d for depletion and expression levels), whereas exogenous expression of a dominant negative form of the deadenylase CAF1B (CAF1B-DDAA) showed inconsistent effects on UPF1 phosphorylation (Fig. 1d). We conclude that interventions that impair steps in the NMD pathway downstream of UPF1 assembly with mRNA substrates, including manipulations of NMD-specific factors as well as general mRNA decay factors, lead to increased UPF1 phosphorylation. We note that a correlation appears to exist between the severity of the NMD defect and the extent of UPF1 phosphorylation, as those of the tested conditions known to most severely inhibit NMD—that is, depletion of SMG6 and mutations of the UPF1 ATPase^10^,^16^,^35^,^36^,^38^,^39^—also cause the largest increase in phosphorylation (Fig. 1a–d).

### UPF1 hyperphosphorylation on inhibition of late NMD steps

**Figure 2:**
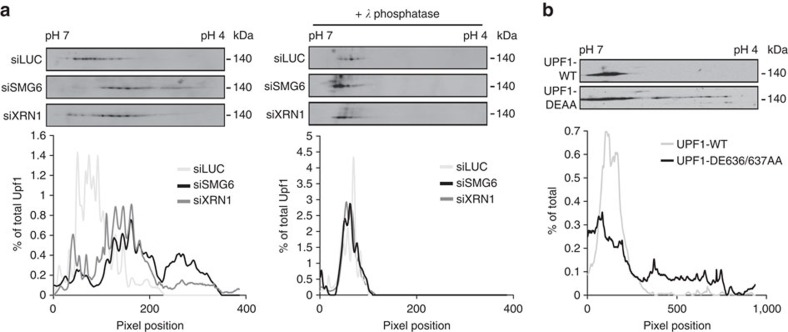
UPF1 is hyperphosphorylated upon SMG6 and XRN1 depletion and upon inhibition of ATPase activity. (**a**) Two-dimensional gel electrophoresis assays performed on extracts from HeLa tet-off cells transfected with siRNAs targeting Firefly Luciferase (siLUC), SMG6 (siSMG6) or XRN1 (siXRN1) and subjected to western blotting with anti-UPF1 antibody. Prior to electrophoresis, cell lysates were treated (+*λ* phosphatase; right panel) or not (left panel) with lambda protein phosphatase. Graphs indicate signal intensity along the blots normalized to the total signal intensity. (**b**) Two-dimensional gel electrophoresis assays performed on extracts from HeLa tet-off cells transfected with plasmids coding for myc-tagged wild-type UPF1 (Upf1-WT) or UPF1 DE636/637AA. The graph indicates signal intensity along the blots normalized to the total signal intensity.

The experiments in Fig. 1 monitored relative levels of UPF1 [S/T]Q phosphorylation, but were not designed to discriminate between an increase in the overall pool of phosphorylated UPF1 versus an increase in the number of phosphorylated residues within individual UPF1 molecules. Moreover, given that the phospho-[S/T]Q antibody could react with some phosphorylation sites more than others, there may not be a linear relationship between the extent of UPF1 phosphorylation and the signal observed in Fig. 1. To address whether UPF1 accumulates in a hyperphosphorylated form upon inhibition of downstream steps in NMD, we performed two-dimensional (2D) gel electrophoresis, as previously used to monitor UPF1 hyperphosphorylation^26^,^40^. As seen in Fig. 2a, depletion of SMG6 or XRN1 resulted in a heterogeneous shift of UPF1 towards lower isoelectric points, as compared with the control condition (left panels, pixel density quantified below, and depletion efficiencies shown in Supplementary Fig. 2). This shift is particularly striking with SMG6 depletion, which also showed the greatest effect in overall phosphorylation levels as monitored in the IP–western blots (Fig. 1a). While the 2D gels do not reveal the exact number of phosphorylations or the extent of phosphorylation at individual sites, the observed change in UPF1 isoelectric points is due to UPF1 hyperphosphorylation as treatment with *λ* phosphatase collapsed the signal and generated UPF1 profiles for SMG6 and XRN1 siRNA-treated cells that were indistinguishable from that of control cells (Fig. 2a, right panels). Similarly, the ATPase-deficient UPF1 DE636/637AA mutant protein showed a shift towards lower isoelectric points as compared with wild-type UPF1 (Fig. 2b). Collectively, our observations demonstrate that disruption of steps in the NMD pathway downstream of UPF1 substrate binding induces UPF1 hyperphosphorylation and that the extent of phosphorylation correlates with the severity of the NMD defect.

### Increase in phospho-UPF1 on ARE-mediated decay activation

**Figure 3:**
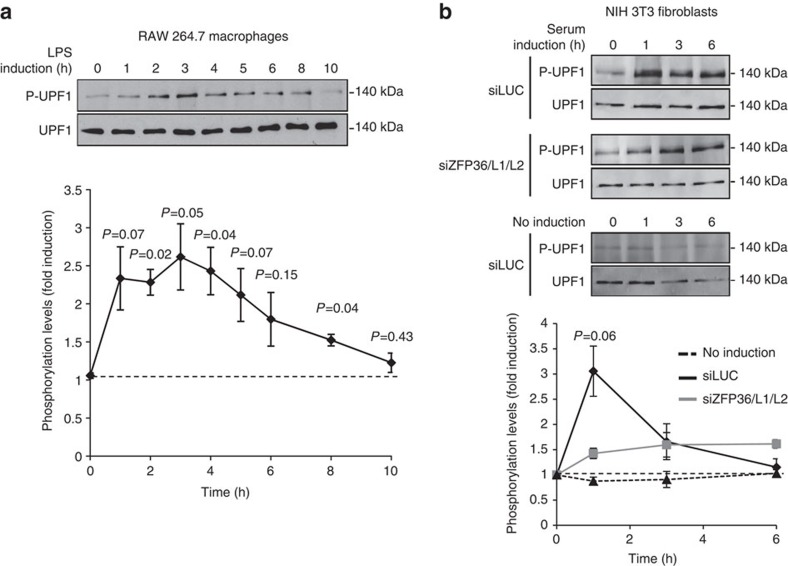
Transient increase in UPF1 phosphorylation upon activation of ARE-mediated decay. (**a**) Western blots showing endogenous UPF1 phosphorylation levels in RAW 264.7 cells treated with LPS and monitored as in Fig. 1a. Numbers refer to hours after LPS addition. Graph showing UPF1 phosphorylation levels quantified as in Fig. 1 is shown below. Indicated values are normalized to the zero time point±s.e.m. from three independent experiments. *P* values are relative to time zero and were calculated using paired two-tailed Student's *t*-test. (**b**) Western blots showing UPF1 phosphorylation levels in NIH 3T3 cells transfected with siRNAs targeting either Firefly Luciferase (LUC) or ZFP36, ZFP36L1 and ZFP36L2 (ZFP36/L1/L2). Numbers above panels refer to time after serum induction; lower panel shows uninduced cells. Graph showing UPF1 phosphorylation levels measured from three independent experiments as in **a** is shown below. *P* value is comparing siLUC to siZFP36/L1/L2 conditions at 1 h after serum induction using the paired two-tailed Student's *t*-test.

Given the observed hyperphosphorylation of UPF1 in response to mRNA decay factor depletion, we predicted that UPF1 might compensate with increased phosphorylation on over-activation of an unrelated mRNA decay pathway that uses RNA decay factors shared with NMD. A well-studied example of a highly induced mRNA decay pathway occurs during an inflammatory response, when a rapid induction in cytokine and chemokine mRNAs is subsequently quenched by activation of the ARE-mediated mRNA decay pathway^41^. This pathway employs several mRNA decay factors also used by NMD^42^,^43^,^44^. As seen in Fig. 3a,b, a transient induction in UPF1 phosphorylation, peaking at 2.5 to 3 times the signal observed for unstimulated cells, occurred on treatment of RAW 264.7 macrophages with lipopolysaccharides (LPS, mimicking bacterial infection; Fig. 3a) and of NIH 3T3 fibroblasts with serum (mimicking injury; Fig. 3b, upper panel). This occurred without an increase in levels of UPF1 (Fig. 3a,b) or of the UPF1 kinase SMG1 (Supplementary Figs 3a,b). The timing of UPF1 phosphorylation induction correlates with the previously described timing of ARE-mediated mRNA decay activation in these cell lines^45^,^46^. Importantly, the transient increase in UPF1 phosphorylation occurs in response to activation of the ARE-mRNA decay pathway as depletion of the responsible mRNA decay activators in NIH 3T3 cells, ZFP36, ZFP36L1 and ZFP36L2, abolished the peak in UPF1 phosphorylation (Fig. 3b), despite the ZFP36/L1/L2-depleted cells responding to serum induction similarly to the control cells as monitored by cFOS expression (Supplementary Fig. 3b). With the caveat that indirect effects on SMG1 activity cannot be ruled out, the simplest interpretation of these observations is that increased UPF1 phosphorylation occurs in response to activation of an mRNA decay pathway that competes for shared mRNA decay machinery.

### Multiple UPF1 [S/T]Q motifs contribute to NMD efficiency

**Figure 4:**
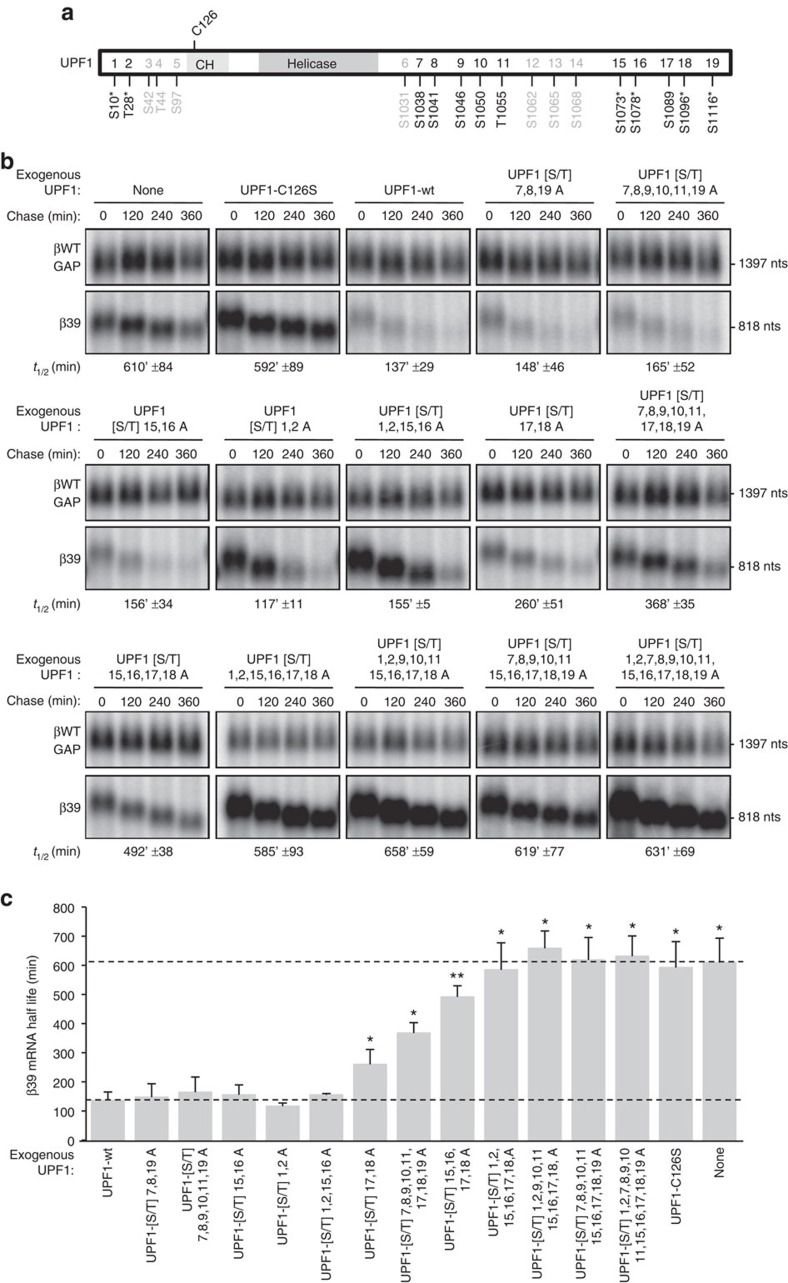
Multiple UPF1 [S/T]Q motifs contribute to NMD. (**a**) Schematic representation of human UPF1 protein showing the amino acids mutated in this study (black); the position of the helicase domain and the cysteine/histidine-rich domain (CH) of UPF1 are indicated (not to scale). Numbers following amino-acid symbols correspond to the position in the UPF1 primary sequence (GenBank #NP_002902). Asterisk indicates confirmed phosphorylated sites. [S/T]Q motifs not investigated in this work are shown in grey. (**b**) Northern blots showing the decay of β39 mRNA in HeLa Tet-Off cells depleted for endogenous UPF1 and expressing myc-tagged variants of UPF1 as indicated. Numbers above panels refer to minutes after tetracycline-mediated transcriptional shutoff of β39 mRNA (chase). β39 mRNA half-lives (*t*_1/2_) were calculated after normalization of levels of β39 mRNA to levels of constitutively transcribed β-globin–GAPDH fusion mRNA (βWT-GAP) and are given as averages±s.e.m. from three independent experiments. Numbers on the right refer to RNA lengths in nucleotides (nts) excluding polyA-tails. (**c**) Graph showing half-lives calculated from experiments presented in **b**. Lower and upper dotted lines represent half-life values for wild-type UPF1 (Upf1-wt) and no add-back (None) conditions respectively. Error bars represent s.e.m. from three independent experiments. *P* values were calculated relative to Upf1-wt conditions using the paired two-tailed Student's *t*-test; *P<0.05 and **P<0.01.

To directly test the contribution of the multiple UPF1 [S/T]Q motifs to UPF1 activity, we constructed plasmids coding for siRNA-resistant versions of UPF1 containing combinations of [S/T]Q to AQ substitutions spanning 12 potential or confirmed phosphorylation sites (Fig. 4a), including sites at positions 28, 1,078, 1,096 and 1,116 that were previously observed to promote association with SMG5-7 proteins^10^,^13^,^17^,^22^,^33^. These mutations are unlikely to affect UPF1 structurally, as the N- and C-terminal regions of UPF1 containing the [S/T]Q motifs are predicted to be unstructured (Supplementary Fig. 4a), and all mutant proteins maintained an unimpaired interaction with UPF2 (Supplementary Fig. 4b). To test the effect of the [S/T]Q to AQ substitutions on UPF1 function, we monitored the ability of UPF1 mutants to complement siRNA-depleted endogenous UPF1 in the degradation of β-globin mRNA containing a PTC at position 39 (β39).

Consistent with phosphorylation at S1096 (here for simplicity labelled position 18; Fig. 4a) playing an important role in NMD, alanine substitutions at positions 17 and 18 caused an increase in the half-life of the β39 mRNA (UPF1 [S/T]17,18A) as compared with that observed with wild-type UPF1 (UPF1-wt; Fig. 4b; quantified in Fig. 4c). Nevertheless, this mutant UPF1 protein was only partially impaired in NMD, supporting degradation of β39 mRNA at a significantly faster rate than that observed in the absence of UPF1 add-back (None), or in the presence of UPF1 C126S, a mutant UPF1 protein that fails to interact with UPF2 (refs 10, 47). No defect in NMD activity was observed for the UPF1 [S/T]1,2A, [S/T]15,16A or [S/T]7,8,19A mutants, which was surprising because phosphorylation at residues T28 (here called position 2), S1078 (position 16) and S1116 (position 19) have previously been shown to support interaction with SMG6 (T28), SMG7 (S1078) and SMG5 (S1116)^10^,^17^,^22^,^33^. Strikingly, combining alanine substitutions that on their own had little or no effect on UPF1 activity, resulted in decreased activity of UPF1 as observed by the increase in β39 mRNA half-lives as [S/T]Q to AQ substitutions were combined, culminating in completely inactivated UPF1 (Fig. 4b,c; compare mutations left to right) despite equal expression of all mutant proteins (Supplementary Fig. 4c). We conclude that none of the 12 tested [S/T]Q motifs are essential for UPF1 function, but multiple [S/T]Q motifs contribute to UPF1 activity with some (such as S1096, and possibly T28, S1078 and S1116) appearing to contribute more than others.

### UPF1 hyperphosphorylation enhances association with SMG5-7

**Figure 5:**
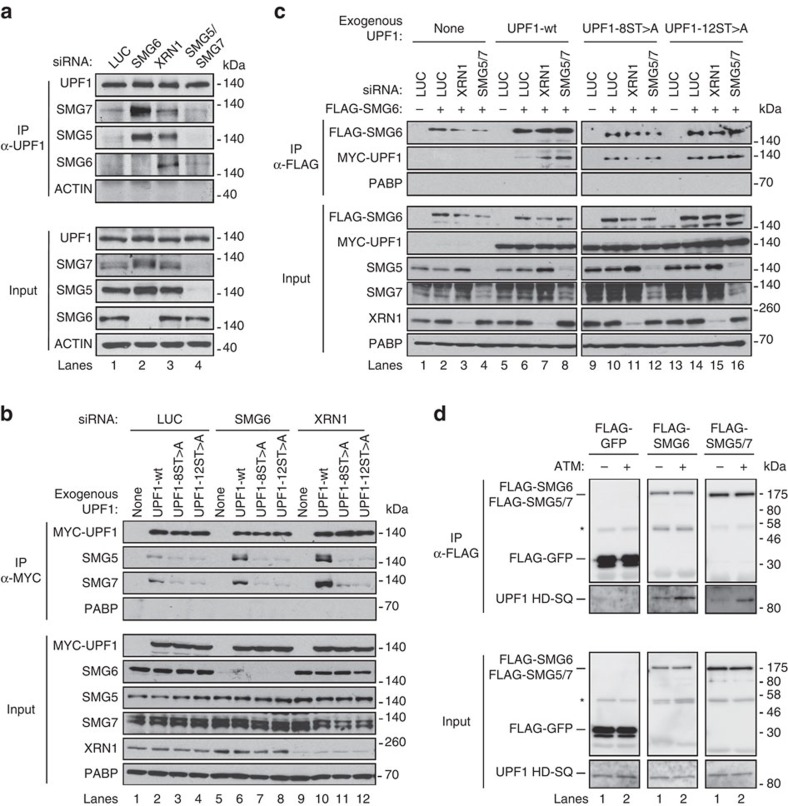
Phosphorylation-dependent enhanced association of UPF1 with SMG5-7 when downstream NMD factors are limiting. (**a**) Western blots showing protein levels in UPF1-immunoprecipitated (IP) or total (1% of Input) fractions from HeLa tet-off cells transfected with the indicated siRNAs. (**b**,**c**) Same as panel **a** but monitoring IPs and input samples for exogenously expressed Myc-tagged wild-type and mutant UPF1 proteins (8ST>A=[S/T]7,8,9,10,11,17,18,19A; 12ST>A=[S/T]1,2,7,8,9,10,11,15,16,17,18,19A). FLAG-tagged SMG6 was co-transfected with Myc-UPF1 in **c**. (**d**) Western blots for *in vitro* pull-downs of bacterially expressed UPF1 HD-SQ with FLAG-tagged GFP, SMG5/7 or SMG6 immuno-isolated from human cells. UPF1 HD-SQ was treated with or without ATM kinase prior to pull-down. Input samples are shown below. *Antibody heavy chain.

What could be the significance of multiple phosphorylation sites contributing to UPF1 function (Fig. 4) and UPF1 undergoing hyperphosphorylation when downstream factors are limiting (Figs 1 and 2)? Given evidence from others that UPF1 is a target of SMG1 only when assembled with mRNA^10^,^22^,^48^, we hypothesized that UPF1 hyperphosphorylation occurs as a consequence of UPF1 stalling on mRNA targets, which in turn allows increased affinity of UPF1 for downstream factors to enhance decay. If so, it is predicted that stalls in the NMD pathway that result in increased UPF1 phosphorylation should lead to increased association of UPF1 with downstream factors in a phosphorylation-dependent manner. Indeed, UPF1 ATP binding and ATPase mutants, which accumulate in hyperphosphorylated forms (Figs 1b and 2b), have previously been observed to assemble more strongly with SMG5-7 than wild-type UPF1 (refs 10, 36). Similarly, as seen in the co-IP assays in Fig. 5a, which were performed in the presence of RNase to eliminate RNA-dependent interactions (Supplementary Fig. 5a), depletion of SMG6 or XRN1 strongly increased complex formation of UPF1 with SMG5 and SMG7 (compare lanes 2, 3 with 1). Moreover, complex formation of UPF1 with SMG6 was enhanced on depletion of XRN1 (lane 3) and, to a lesser extent, of SMG5/7 (lane 4). These observations show that manipulations that impair the NMD pathway downstream of UPF1 mRNA substrate binding result in increased RNA-independent association of UPF1 with downstream SMG5-7 factors.

To test whether the observed increase in association of UPF1 with downstream factors is dependent on UPF1 phosphorylation, we compared the extent of SMG5-7 complex formation for UPF1 wild-type with two of the UPF1 [S/T]Q mutants: UPF1 [S/T]7,8,9,10,11,17,18,19A (labelled UPF1-8ST>A in Fig. 5b), which is partially defective for NMD, and UPF1 [S/T]1,2,7,8,9,10,11,15,16,17,18,19A (UPF1-12ST>A), which is fully defective for NMD (Fig. 4). As seen in Fig. 5b, in contrast to wild-type UPF1 (lanes 2, 6 and 10), the UPF1 [S/T]Q mutants fail to acquire enhanced association with SMG5 and SMG7 on depletion of SMG6 or XRN1 and instead maintain low level of SMG5 and SMG7 association similar to that observed in the absence of SMG6 or XRN1 depletion (compare lanes 7, 8, 11, 12 with 3, 4). Similarly, as seen in Fig. 5c, wild-type and [S/T]Q mutant UPF1 can all be observed to associate with SMG6 (lanes 5-16), but only wild-type UPF1 shows enhanced association with SMG6 on depletion of XRN1 or SMG5/SMG7 (lanes 6–8). Thus, UPF1 appears to exhibit a basal level of affinity for SMG5-7 proteins that is independent of hyperphosphorylation, consistent with recent observations for SMG6 (refs 32, 49), which is further stimulated by phosphorylation. This was further supported by *in vitro* pull-down assays, in which bacterially produced UPF1 was observed to associate with SMG5/7 and SMG6 in a manner enhanced by phosphorylation with recombinant SQ-specific ATM kinase (Fig. 5d). Collectively, the observations in Fig. 5 demonstrate UPF1 hyperphosphorylation as a mechanism for enhancing the affinity of UPF1 for SMG5-7 proteins during a stall in the degradation step of the NMD pathway.

### UPF1 hyperphosphorylation importance upon SMG5/7 depletion

**Figure 6:**
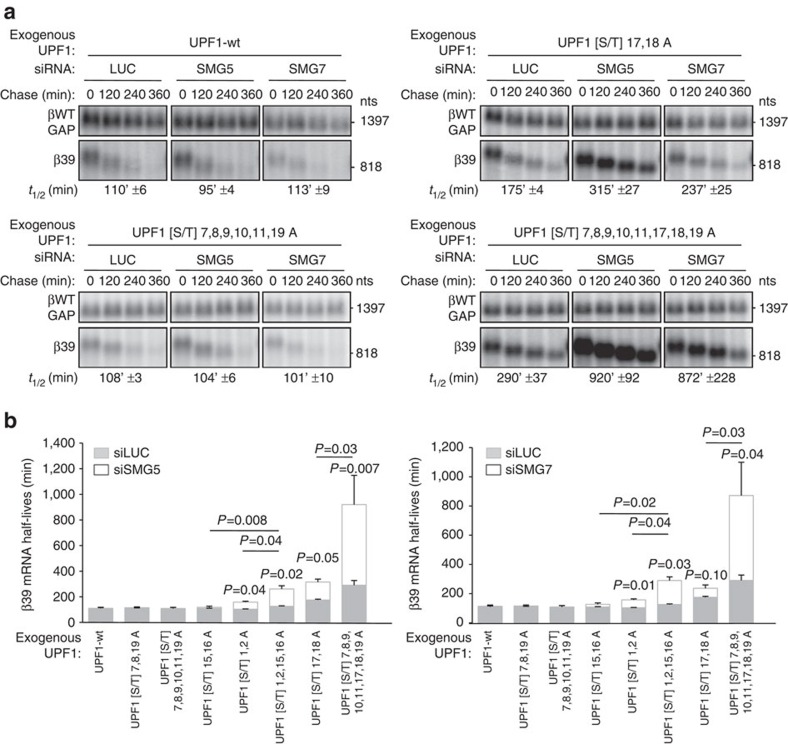
UPF1 hyperphosphorylation compensates for SMG5 and SMG7 depletion. (**a**) Northern blots showing the decay of β39 mRNA in HeLa Tet-Off cells depleted for endogenous UPF1 and expressing the indicated exogenous variants of UPF1. In addition, cells were treated with siRNAs targeting SMG5 or SMG7, or as a control, Firefly Luciferase (LUC). Numbers above the panels refer to minutes after tetracycline-mediated transcriptional shutoff of β39 mRNA (chase). β39 mRNA half-lives (*t*_1/2_) were calculated from three independent experiments as described for Fig. 4b. Numbers on the right refer to RNA lengths in nucleotides (nts) excluding polyA-tails. (**b**) Graphs showing β39 mRNA half-lives calculated from mRNA decay assays presented in **a** and Supplementary Fig. 6a. Graphs compare siLUC control conditions (grey) to siSMG5 (white; left graph) or siSMG7 conditions (white; right graph). Error bars represent s.e.m. from three independent experiments. *P* values indicated above bars compare siSMG7 and siSMG5 conditions to control siLUC. *P* values indicated above brackets compare siSMG7 and siSMG5 conditions between different UPF1 mutants (all *P* values were calculated using the paired two-tailed Student's *t*-test).

If UPF1 hyperphosphorylation serves to enhance the affinity of UPF1 for downstream factors on stalls in the NMD pathway, then the ability of UPF1 to undergo hyperphosphorylation should become increasingly important for NMD as the availability of downstream factors is limited. Indeed, as seen in the mRNA decay assays in Fig. 6a and Supplementary Fig. 6a, while low-level depletion of SMG7 or SMG5 did not reduce the efficiency of NMD in the presence of wild-type UPF1 (Fig. 6a, top left panel), many of the UPF1 [S/T]Q mutants became impaired in their NMD activity under these conditions (quantified in Fig. 6b, compare white to grey bars) despite comparable SMG5/7 depletion efficiencies (Supplementary Fig. 6b). This impairment in NMD efficiency on SMG5 or SMG7 depletion became increasingly pronounced as groups of [S/T]Q to AQ mutations were combined (compare individual mutations in Fig. 6a and in Supplementary Fig. 6a; *P* values, calculated using the paired two-tailed Student's *t*-test, are indicated in Fig. 6b). Thus, the ability of UPF1 to undergo hyperphosphorylation becomes increasingly important for NMD as downstream factors SMG5 or SMG7 are rendered limiting, consistent with UPF1 hyperphosphorylation playing an important role in rescuing slow kinetics during the degradation phase of the NMD pathway. Collectively, our findings suggest UPF1 hyperphosphorylation as an important mechanism for the NMD pathway to sense and overcome limitations in downstream factors including NMD-specific factors as well as general mRNA decay machinery.

## Discussion

**Figure 7:**
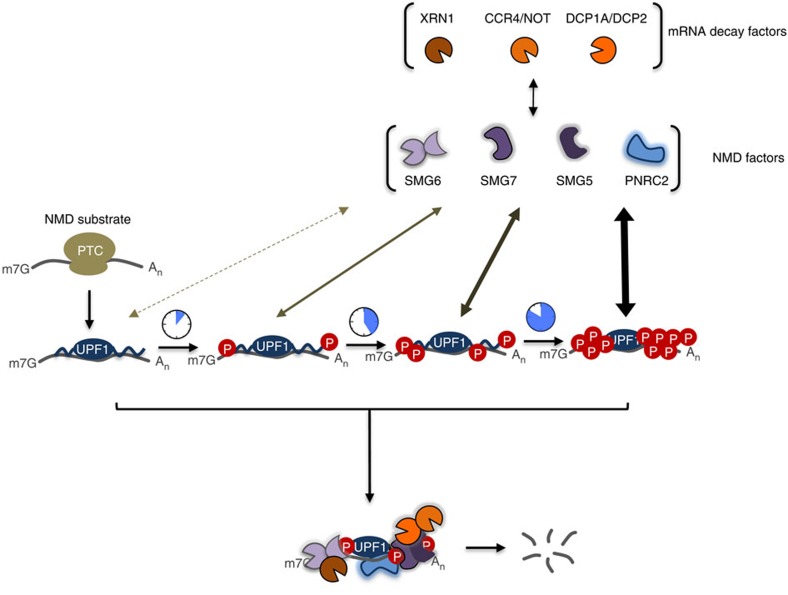
Model representing the amplification of mRNA decay signal through UPF1 hyperphosphorylation. When downstream decay steps are limiting, UPF1 stalls on NMD-targeted mRNPs and is progressively phosphorylated on [S/T]Q motifs. The increase in phosphorylation progressively promotes the ability of UPF1 to activate mRNA decay.

Phosphorylation at specific sites within UPF1, the central factor in NMD, was known to stimulate the association of UPF1 with downstream SMG5-7 factors^10^,^13^,^22^,^35^,^48^, but why UPF1 contains multiple phosphorylation sites, most of which are conserved over evolution (Supplementary Fig. 1a) has been unclear. Here we present evidence that no single phosphorylation site is essential for UPF1 function, but multiple phosphorylation sites contribute to UPF1 activity with individual sites contributing to different extents, as evidenced by the cumulative effects on UPF1 activity of mutations in phosphorylation sites (Fig. 4). Stalls in the NMD pathway caused when NMD-specific or general mRNA decay factors are rendered limiting result in hyperphosphorylation of UPF1 (Figs 1 and 2) and in phosphorylation-dependent increased affinity of UPF1 for downstream SMG5-7 factors (Fig. 5). The ability of UPF1 to undergo hyperphosphorylation becomes increasingly important for NMD when downstream SMG5 or SMG7 NMD factors are limited (Fig. 6). Taken together, these observations suggest a mechanism by which UPF1 hyperphosphorylation serves as a molecular clock to render UPF1 increasingly proficient at activating the mRNA decay machinery with time of residence on target mRNAs (Fig. 7), an idea consistent with the specificity of the SMG1 kinase for mRNA-associated UPF1 (refs 10, 22, 48). This hyperphosphorylation mechanism might play an important role in preventing UPF1 from activating degradation of non-target mRNAs, which we recently found transiently associate with UPF1 (ref. 24). It might also ensure that the NMD pathway can rid the cell of potentially deleterious aberrant mRNAs no matter current demands on cellular mRNA decay machineries. Consistent with the latter idea, manipulations that transiently activate the ARE-mediated mRNA decay pathway in fibroblast and macrophage cell lines triggered a transient spike in UPF1 phosphorylation, which was dependent on the central ARE-mRNA decay factors (Fig. 3).

How does a stall in mRNA degradation trigger UPF1 hyperphosphorylation? The simplest explanation is that UPF1 is phosphorylated by SMG1 specifically when assembled in an mRNP complex, resulting in progressively increased phosphorylation of UPF1 as the NMD-mRNP is awaiting degradation (Fig. 7). This is consistent with previous studies suggesting that UPF1 is a target of SMG1 only when assembled with mRNA^10^,^22^,^48^. Indeed, previous studies found SMG1 locked in a catalytic inactive state by SMG8 and SMG9 co-factors until the SMG1/8/9 complex is recruited to NMD-targeted mRNPs^50^,^51^, and UPF1 phosphorylation is dependent on complex formation with UPF2 and translation release factors^10^.

How might increased UPF1 phosphorylation lead to increased UPF1 activity? Our observations suggest that UPF1 hyperphosphorylation results in increased affinity of UPF1 for downstream SMG5-7 factors (Fig. 5), which in turn would increase the ability of the resulting UPF1-cofactor complex to activate mRNA decay (Fig. 7). Consistent with this, evidence has been presented for phosphorylation of UPF1 at specific sites promoting association with SMG5-7 proteins^10^,^17^,^33^. PNRC2, which has been shown to link UPF1 with the DCP2 decapping complex, has also been observed to associate preferentially with phosphorylated UPF1 (ref. 15). Our observations that none of the phosphorylation sites currently known to interact with SMG5-7 proteins are essential for NMD and that additional sites contribute to NMD efficiency (Fig. 4), suggest that multiple phosphorylation sites can support recruitment of downstream factors in the pathway. It is also a possibility that increased phosphorylation of UPF1 promotes downstream steps in the NMD pathway beyond factor recruitment. For example, in addition to stimulating initiating events in mRNA degradation via endonucleolytic cleavage, decapping or deadenylation, UPF1 phosphorylation could also promote the downstream exonucleolytic degradation of the mRNA body carried out by general exonucleases. Consistent with this, depletion of the general 5′-to-3′ exonuclease XRN1, required for degrading endonucleolytically cleaved as well as decapped decay intermediates, induced an increase in UPF1 phosphorylation (Figs 1 and 2) and in phosphorylation-dependent UPF1 association with NMD factors (Fig. 5).

The importance of UPF1 hyperphosphorylation is likely conserved in eukaryotes given the conservation of UPF1 hyperphosphorylation and of UPF1 [S/T]Q motifs in metazoans^13^,^19^,^28^ (Supplementary Fig. 1a). Even in *S. cerevisiae* where no SMG1 homologue has been identified, multiple UPF1 phosphorylation sites have recently been described^27^. An important question for future study is whether a hierarchy exists between UPF1 phosphorylation sites. For example, the rate and/or order of phosphorylation could differ between individual sites, and the ability of individual sites to recruit and/or activate individual downstream factors could vary. Consistent with this idea, some [S/T]Q motifs appear to be more important than others for UPF1 activity (Figs 4 and 6). Moreover, an interesting question is how the rapid phosphorylation-dependent buffering of the NMD pathway uncovered in this study is integrated with the longer-term autoregulatory mechanism that was recently revealed, whereby central factors in the NMD pathway are upregulated when NMD is limiting^52^,^53^. In addition to promoting efficient mRNA degradation, the UPF1 hyperphosphorylation mechanism could also serve a function as a checkpoint to ensure that UPF1 is correctly associated with a target mRNA before activation of degradation, thereby preventing spurious degradation of non-target mRNAs with which UPF1 associates transiently^24^. However, it seems unlikely that a specific phosphorylation threshold exists upon which the NMD pathway is activated, given that the extent of UPF1 phosphorylation varies with the severity of the impairment of the NMD pathway (Figs 1 and 2, and Supplementary Fig. 1b), and that the number of UPF1 phosphorylation sites becomes increasingly important for NMD as downstream factors are rendered limiting (Fig. 6).

Reversible post-translational modifications are common in regulatory proteins involved in gene expression, but while their importance in chromatin regulation has long been acknowledged, their role in mRNA regulation remains poorly understood^54^. Our observations reveal a role for hyperphosphorylation of an RNA-binding protein as a mechanism for its associated mRNP to increasingly activate mRNA decay over time. Repetitive phosphorylation events are common features of central factors in gene expression. For example, RNA polymerase II contains in its C-terminal domain a large array of tandemly repeated heptameric peptides (for example, 52 in humans), each containing phosphorylation sites for a key set of kinases important for recruitment of elongation and processing factors^55^,^56^,^57^,^58^,^59^,^60^,^61^. Similarly, SR proteins, which play critical roles in pre-mRNA splicing, contain multiple SR repeats that serve as phosphorylation sites for SR kinases^62^,^63^. Thus, the principle of hyperphosphorylation as a feedback mechanism to increase affinity for downstream factors in response to stalling might be applicable also to other processes in gene expression, for example in the competition between genes for transcription elongation or pre-mRNA processing factors or between pre-mRNAs for the splicing machinery.

## Methods

### Cell cultures

In all experiments, HeLa tet-off (Clontech), RAW 264.7 (ATCC) and NIH 3T3 tet-off (Clontech) cells were grown in DMEM (Gibco) with 10% heat-inactivated fetal bovine serum (FBS, Gibco). When indicated, RAW 264.7 cells were treated with 100 ng ml^−1^ lipopolysaccharide (LPS; Sigma-Aldrich, L6529). NIH 3T3 tet-off cells were synchronized in G0 by growing cells for 48 h in DMEM with 0.2% heat-inactivated FBS, followed by replacement of media with DMEM containing 20% heat-inactivated FBS (Gibco).

### Plasmid constructs and siRNA

pcMyc-UPF1 [S/T]Q to AQ plasmids, pcMyc-UPF1 G495R, pcMyc-UPF1 G497E and pcMyc-UPF1-C126S were generated from a pcMyc-UPF1 construct based on pcDNA3 containing full-length Upf1 (amino acids 1–1,118) with an N-terminal Myc-tag by using the quick change site-directed mutagenesis method (Stratagene). pcFlag-CAF1B DDAA, pcFlag-DCP2 E148Q, pcDNA-myc-UPF1 DE636/637AA and pcDNA-myc-UPF1 K498A have been previously described^36^,^64^,^65^. pPC-β39 and pcβWT-UAC-GAP used in pulse-chase experiments have been described earlier^66^. The following siRNAs were used in this study (only sense strand is listed): HEDLS siRNA; 5′- GAGUUAAAGAUGUGGUGUA -3′; LUC siRNA:5′- CGUACGCGGAAUACUUCGA -3′; PNRC2 siRNA: 5′- UUGGAAUUCUAGCUUAUCA -3′ (ref. 15); SMG5 siRNA: 5′- GCCAGAAAGAGGUGGGAAA -3′ (ref. 14); SMG6 siRNA: 5′- GCUGCAGGUUACUUACAAG -3′ (ref. 16); SMG7 siRNA: 5′- GCAAGAAACAUCUGUGAUA -3′ (ref. 14); UPF1 siRNA: 5′- CCAAGAUGCAGUUCCGCUCCA -3′ (ref. 67); XRN1 siRNA: 5′- AGAUGAACUUACCGUAGAA -3′ (ref. 16); ZFP36 siRNA: 5′- GAAUCCUGGUGCUCAAAUU -3′; ZFP36L1 siRNA: 5′- CCACAACUCAAUAUGAAAA -3′; ZFP36L2 siRNA: 5′- GUAACAAGAUGCUCAACUA -3′.

### Immunoprecipitation assays

For experiments involving siRNA-mediated depletions, cells were transfected with 20 nM siRNAs using siLentFect (Bio-Rad) according to manufacturer's recommendations. Forty-eight hours after the first siRNA transfection, cells were transfected a second time using the same conditions. Twenty-four hours later, cells were collected in 1 ml of PBS solution (8 g l^−1^ NaCl, 0.2 g l^−1^ KCl, 1.44 g l^−1^ Na_2_HPO_4_, 0.24 g l^−1^ KH_2_PO_4_, pH 7.4). In UPF1/SMG6 co-immunoprecipitation assays, 200 ng of pcDNA-myc-UPF1 or pcMyc-UPF1 [S/T]Q mutants and 500 ng of pcDNA-Flag-SMG6 or pcFlag were transfected using TransIT HeLa Monster reagent according to the manufacturer's protocol (Mirus). In experiments involving expression of dominant negative proteins, 200 ng of pcMyc-UPF1-wt, 0.5 μg of pcFlag-CAF1B DDAA, 0.5 μg of pcFlag-DCP2 E148Q and 0.5 μg of empty pcFlag plasmid were transfected using TransIT HeLa Monster reagent according to the manufacturer's protocol (Mirus). Forty-eight hours after transfection, cells were harvested in 1 ml PBS. For measuring phosphorylation of UPF1 ATPase mutants, 200 ng of pcMyc-UPF1-wt or 200 ng of pcMyc-UPF1 mutants (DE636/637AA, K498A, G495R and G497E) were transfected with 0.8 μg of empty pcFlag plasmid using TransIT HeLa Monster reagent according to the manufacturer's protocol. Forty-eight hours after transfection, cells were collected in 1 ml PBS by scraping. Cells collected in PBS were then pelleted and lysed in 400 μl of hypotonic gentle lysis buffer (10 mM Tris-HCl pH 7.5, 10 mM NaCl, 2 mM EDTA, 0.5% Triton X-100, 0.5 mM PMSF, 2 mg ml^−1^ leupeptin, 2 mg ml^−1^ aprotinin and 1 × PhosphataseArrest I phosphatase inhibitor cocktail (GBiosciences)) for 5 min on ice. NaCl concentration was brought to 150 mM and, in co-IP experiments in Fig. 5, RNase A was added at 125 μg ml^−1^. Lysates were incubated for 4 h with 50 μl of protein A sepharose CL-4B beads (GE Healthcare Life Sciences) conjugated to 2.5 μl of rabbit polyclonal anti-UPF1 antibody^68^ or 2.5 μl of mouse monoclonal anti-Myc (Cell Signaling, clone 9B11). Beads were then washed seven times with NET2 (50 mM Tris-HCl pH 7.5, 150 mM NaCl and 0.05% Triton X-100) and resuspended in 30 μl of 2X SDS loading buffer (100 mM Tris-HCl pH 6.8, 4% SDS, 12% β-mercaptoethanol, 20% glycerol and 0.1% bromophenol blue). Immunoprecipitates were separated by SDS–PAGE followed by western blotting.

### *In vitro* kinase assays

Bacterially expressed and purified UPF1-HD-SQ^69^ was incubated with 150 ng of recombinant ATM (Millipore 14-933), 10  μM ATP (+20 μCi γ^32^P-ATP in assays shown in Supplementary Fig. 5b) in 1 × kinase buffer (10 mM HEPES pH 7.5, 50 mM NaCl, 10 mM MgAc, 10 mM MnCl_2_, 20 mM β-glycerophosphate and 1 mM DTT) 30 min at 30 °C. *In vitro* kinase reactions were then analysed by sodium dodecyl sulfate (SDS)-polyacrylamide gel electrophoresis or used in pull-down assays.

### *In vitro* pull-down assays

Five microgram of pSG5-FLAG GFP (generously donated by Dr Samuel Buchsbaum) or 5 μg of pcDNA-FLAG-SMG5 and 5 μg of pcDNA-FLAG-SMG7 or 5 μg of pcDNA-FLAG-SMG6 and 10 nM UPF1 siRNA were transfected in 10 cm dishes using jetPRIME (Polyplus) according to the manufacturer's protocol. Forty-eight hours later, cells were collected in PBS and lysed in 800 μl of hypotonic gentle lysis buffer (10 mM Tris-HCl pH 7.5, 10 mM NaCl, 2 mM EDTA, 0.5% Triton X-100, 1 × protease inhibitor cocktail (cOmplete Tablets EDTA-free, Roche) for 5 min on ice. NaCl concentration was brought to 150 mM and RNase A was added at 125 μg ml^−1^. Lysates were incubated for 2 h with 100 μl of anti-FLAG M2 affinity gel (Sigma-Aldrich). Beads were then washed seven times with NET2 (50 mM Tris-HCl pH 7.5, 150 mM NaCl and 0.05% Triton X-100) and resuspended in 400 μl of NET2 containing 1 × protease inhibitor cocktail (cOmplete Tablets EDTA-free, Roche), 20 mM β-glycerophosphate and UPF1-HD-SQ *in vitro* kinase reactions. After 2 h incubation at 4 °C, beads were washed five times in NET2 and resuspended in 30 μl of 2 × SDS loading buffer (100 mM Tris-HCl pH 6.8, 4% SDS, 12% β-mercaptoethanol, 20% glycerol and 0.1% bromophenol blue). Immunoprecipitates were separated by sSDS–PAGE followed by western blotting.

### Antibodies

Western blotting were performed using the following antibodies: rabbit polyclonal anti-Myc (Sigma-Aldrich, C3956; 1:1,000), rabbit polyclonal anti-FLAG (Sigma-Aldrich, F7425; 1:1,000), rabbit polyclonal anti-ACTIN (Cell Signaling, 4967S; 1:1,000), mouse monoclonal anti-Myc (Cell Signaling, 9B11; 1:1,000), rabbit polyclonal anti-CBP80 (generously provided by E. Izaurralde; 1:1,000), rabbit polyclonal anti-SMG5 (36; 1:1,000), rabbit polyclonal anti-SMG7 (36; 1:1,000), rabbit polyclonal anti-SMG6 (Abcam ab87539; 1:1,000), rabbit polyclonal anti-ZFP36 N terminus (Sigma-Aldrich, T5327; 1:500), rabbit polyclonal anti-ZFP36L1 (Cell Signaling, 2119S, 1:1,000), mouse monoclonal anti-ZFP36L2 (Santa Cruz Technologies, sc-365908, 1:1,000), mouse monoclonal anti-HuR (Santa Cruz Technologies, sc-5261; 1:20,000), rabbit polyclonal anti-cFOS (Santa Cruz Technologies, sc-52; 1:1,000), rabbit polyclonal anti-SMG1 (Abcam, ab30916; 1:1,000), rabbit polyclonal anti-SIN3A (Millipore, 06–913; 1:1,000), rabbit monoclonal anti-GAPDH (Cell Signaling, 14C10; 1:1,000), rabbit polyclonal anti-XRN1 (ref. 44; 1:500), rabbit polyclonal anti-DCP1A (ref. 44; 1:1,000), rabbit polyclonal anti-UPF1 (ref. 68; 1:1,000), rabbit polyclonal anti-UPF2 (ref. 68; 1:1,000) and rabbit polyclonal anti-HEDLS (ref. 70; 1:1,000). UPF1 phosphorylation measurements were performed by incubating blots simultaneously with mouse polyclonal anti-UPF1 (ref. 68; 1:1,000) and rabbit polyclonal anti-Phospho-(Ser/Thr) ATM/ATR Substrate (Cell Signaling, 2851; 1:500) or rabbit polyclonal anti-Phospho-S1127-UPF1 (Millipore, 07–1016; 1:1000). Western blots quantifications were performed either by using Cy3 or Alexa488 conjugated secondary antibodies or by using horseradish peroxidase-coupled secondary antibodies in combination with ECL plus western blot substrate (Thermo Scientific). Blots were then visualized using a PhosphorImager (Typhoon Trio; Amersham Biosciences). Quantifications were performed using ImageQuant TL 1D software. UPF1 phosphorylation levels were determined by normalizing the anti-Phospho-(Ser/Thr) ATM/ATR Substrate signal to the anti-UPF1 signal. *P* values were calculated by performing paired two-tailed Student's *t*-test.

### Two-dimensional gel electrophoresis

Cells were either transfected with 20 nM siRNAs targeting Firefly Luciferase, SMG6 or XRN1 or with 1 μg of pcMyc-UPF1-wt or pcMyc-UPF1 DE636/637AA as described in the immunoprecipitation section above. Cells were then collected in 5 ml of PBS, pelleted and lysed 10 min on ice in 150 μl hypotonic gentle lysis buffer (see above) with or without 1 × PhosphataseArrest I phosphatase inhibitor cocktail (G Biosciences). Cleared lysates were incubated 2 hours at 30 °C with or without 2,000 units of Lambda Protein Phosphatase according to manufacturer's protocol (New England Biolabs). Samples were then incubated in a total of 1 ml of 2D sample buffer (2% CHAPS, 100 mM iodoacetamide, 0.002% bromophenol blue, 8 M urea, 50 mM dithiothreitol) for 10 min at room temperature. 2 M thiourea was added to cells for 10 min and samples were deionized 1 h with 50 mg of AG 501-X8-(D) beads (Bio-Rad). 200 μl of samples were complemented with 1 × ampholytes (Bio-Rad) and separated in ZOOM strip pH 4–7 strips according to manufacturer's protocol (Life Technologies). Strips were then equilibrated for 10 min at room temperature in equilibration buffer 1 (0.375 M Tris-HCl pH 8.8, 6 M urea, 2% sodium dodecyl sulfate, 20% glycerol, 2% dithiothreitol) and 10 min at room temperature in equilibration buffer 2 (0.375 M Tris-HCl pH 8.8, 6 M urea, 2% SDS, 20% glycerol and 135 mM iodoacatamide). Second-dimension electrophoresis was performed in 10% SDS–polyacrylamide gels. Western blots quantifications were performed by using horseradish peroxidase-coupled secondary antibodies in combination with ECL plus western blot substrate (Thermo Scientific). Blots were then visualized using a PhosphorImager (Typhoon Trio; Amersham Biosciences). Quantifications were performed using ImageQuant TL 1D software. Each pixel was attributed an x value corresponding to pixel positions along the first-dimension electrophoresis (pixel position). The pixel intensity for each value of x was normalized by the sum of all pixel intensities.

### Pulse-chase mRNA decay assays

HeLa Tet-Off cells were grown in DMEM (Gibco) supplemented with 10% heat-inactivated FBS (Gibco) and 50 ng ml^−1^ of tetracycline (Sigma-Aldrich). For each well of 12-well plates, cells were transfected with 20 nM siRNA targeting UPF1, and with 20 nM siRNAs targeting luciferase, SMG5 or SMG7 as indicated, using siLentFect (Bio-Rad) according to manufacturer's recommendations. Twenty-four hours after siRNA transfection, cells were transfected using TransIT HeLa Monster reagent (Mirus) according to the manufacturer's protocol, with 0.8 μg of tetracycline-inducible plasmid pPC-β39, 40 ng of internal control pcβWT-UAC-GAP^44^ and 100 ng of pcMyc-UPF1-wt, pcMyc-UPF1 mutants or empty pcDNA-Myc plasmids. Twenty-four hours after plasmid transfection, cells were transfected a second time with 20 nM siRNA targeting UPF1 using siLentFect (Bio-Rad). Twenty-four hours later, transcription was pulsed by washing cells with PBS and adding DMEM with 10% FBS without tetracycline. Six hours after removal of tetracycline, 1 μg ml^−1^ of tetracycline was added to repress transcription. Starting 30 min later (defined as time 0), cells in individual wells were collected in 500 μl Trizol (Ambion) at time points indicated in individual experiments and total RNA isolation was performed according to the manufacturer's protocol. RNA samples were analysed by electrophoresis (1.2% agarose, 6% formaldehyde and 0.02 M 3-(*N*-morpholino)propanesulfonic acid) followed by transfer on Zeta-Probe nylon membrane (Bio-Rad) in 10 × SSC (1.5 M NaCl, 0.15 M sodium citrate, pH 7)^68^. Blots were incubated overnight at 60 °C in hybridization buffer (50% deionized formamide, 2 × SSC, 0.5% SDS, 25 mM sodium phosphate buffer pH 6.5, 3 mg total yeast RNA and 5 × Denhardt's reagent) containing ^32^P-αUTP-labelled β-globin antisense RNA probe, washed four times with wash buffer (0.1 × SSC and 0.1% SDS) and then visualized and quantified using a PhosphorImager (Typhoon Trio; Amersham Biosciences) and ImageQuant TL 1D software. *P* values were calculated by performing paired two-tailed Student's *t*-test.

### qRT-PCR

Cells transfected with 20 nM siRNA targeting either LUC or PNRC2 were collected in 500 μl of TRIzol (Life Technologies) and RNA was isolated according to the manufacturer's protocol. 1 μg of total RNA was reverse-transcribed with 100 ng of eight nucleotide random primers using SuperScript III Reverse Transcriptase (Life Technologies) as recommended by the manufacturer's protocols. PCRs were performed on one one-hundredth of the reverse transcription (RT) reaction using Fast SYBER Green Master Mix (Life Technologies) according to manufacturer's recommendations and the Applied Biosystems StepOnePlus Real-Time PCR System technology. Primers used for PCR: GAPDH: forward, 5′- ATTTGGTCGTATTGGGCGCCTG -3′; reverse, 5′- AGCCTTGACGGTGCCATGGAATTT -3′; PNRC2: forward, 5′- AGATGGGTGGTGGAGAGAGG -3′; reverse, 5′- TTGCATGGCCTGCCAGGCAG -3′. Each measurement was performed in duplicate to calculate the average C_t_ (Threshold Cycle) value. For each set of primers and each PCR assay, a 10-fold standard dilution was performed to calculate the PCR efficiency (*E*). When *E* value ranged between 1.8 and 2.1, mRNA levels were determined using average *C*_t_ value and *E*. PNRC2 mRNA levels were normalized to GAPDH mRNA levels. *P* values were calculated using a two-tailed paired Student's *t*-test.

Blots have been cropped for presentation purposes. Full-length versions are available in Supplementary Fig. 7.

### Data availability

The authors declare that all other relevant data supporting the findings of this study are available on request.

## Additional information

**How to cite this article**: Durand, S. *et al*. Hyperphosphorylation amplifies UPF1 activity to resolve stalls in nonsense-mediated mRNA decay. *Nat. Commun.* 7:12434 doi: 10.1038/ncomms12434 (2016).

## Supplementary Material

Supplementary Information Supplementary Figures 1-7 and Supplementary Reference
