# Supplementary material for: Hyperphosphorylation amplifies UPF1 activity to resolve stalls in nonsense-mediated mRNA decay
Source: Nat Commun. 2016 Aug 11;7:12434. doi: 10.1038/ncomms12434 (PMC4987530; doi:10.1038/ncomms12434)
Supplement: Supplementary Information — Supplementary Figures 1-7 and Supplementary Reference [file ncomms12434-s1.pdf]

Supplementary Figure 1

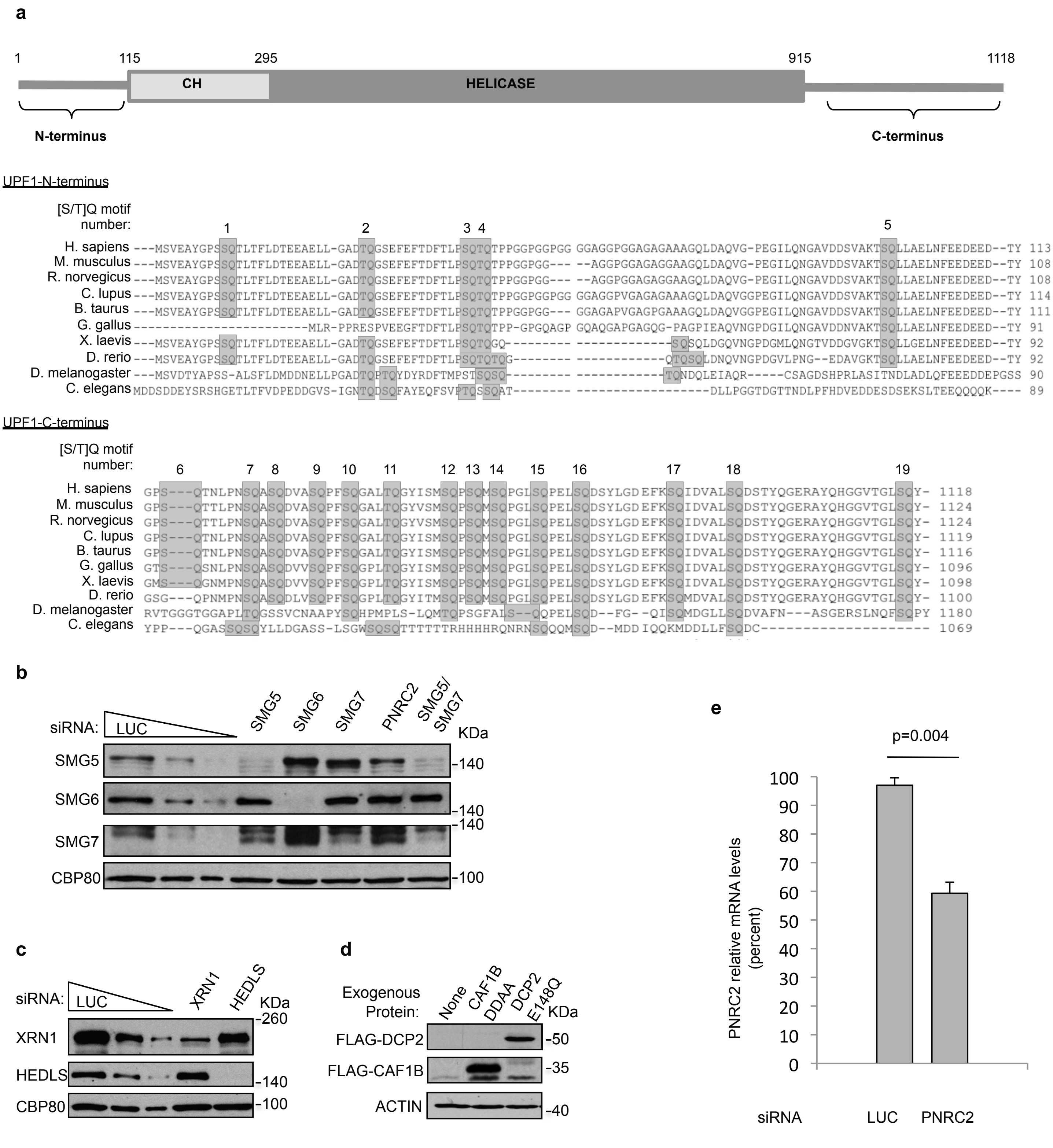

Supplementary Figure 1

(**a**) Alignment of UPF1 N- and C-terminal sequences in different eukaryotes indicated on the left. Alignments were performed with ClustalW2 (http://www.ebi.ac.uk/Tools/msa/clustalw2/). Grey boxes indicate [S/T]Q motifs. A schematic representing UPF1 is shown above; CH: Cystein/Histidine-rich domain. (**b**) Western blot monitoring protein levels in HeLa tet-off cells transfected with the indicated siRNAs. The three first lanes represent a 3 fold serial dilution of the LUC siRNA-transfected lysate. (**c**) Same as in *b*. (**d**) Western blots monitoring protein levels in HeLa tet-off cells transfected with indicated plasmids. (**e**) Quantitative (q)RT-PCR monitoring PNRC2 mRNA levels in experiments in Fig. 1A. All commercially available antibodies we tested failed to detect PNRC2 in Western Blot.

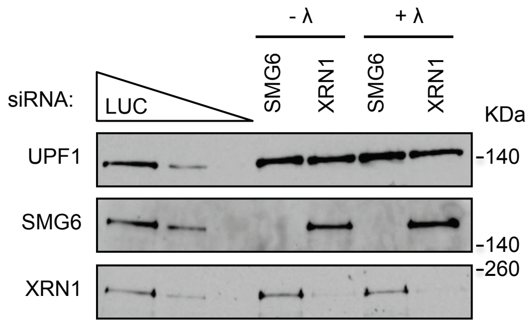

Supplementary Figure 2

Western blot monitoring protein levels in HeLa tet-off cells transfected with the indicated siRNAs used in two-dimensional gel electrophoresis assays in Figure 2a. The three first lanes represent a 3 fold serial dilution of the LUC siRNA-transfected control lysate.

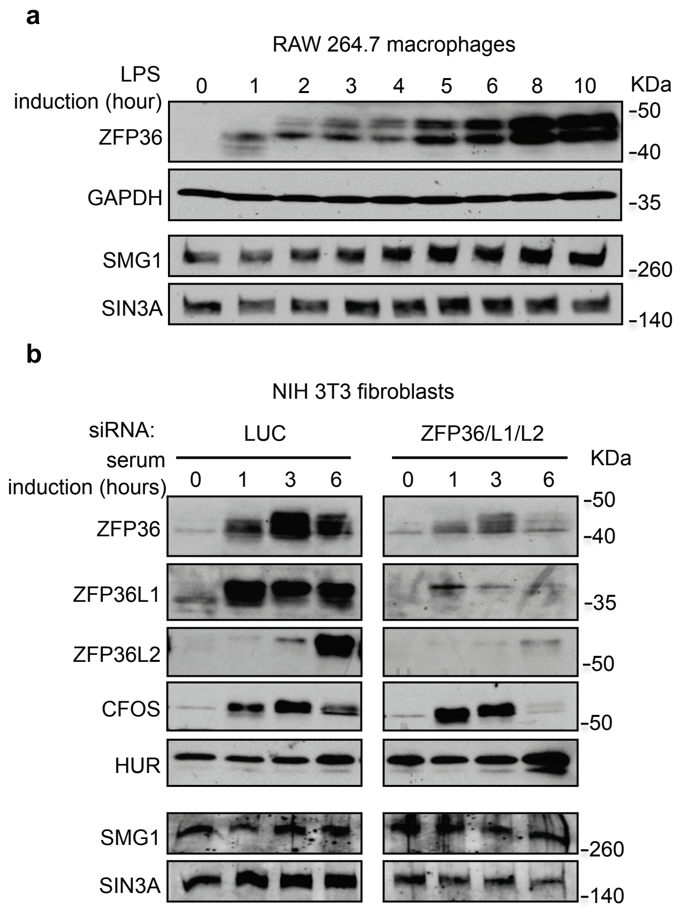

Supplementary Figure 3

(**a**) Western blots showing protein levels in RAW 264.7 cells treated with LPS. Numbers refer to hours after LPS treatment. (**b**) Western blots showing protein levels in NIH 3T3 cells transfected with siRNA targeting Firefly Luciferase (LUC) or ZFP36, ZFP36L1 and ZFP36L2 (ZFP36/L1/L2). Numbers above panels refer to hours after serum induction.

Supplementary Figure 4

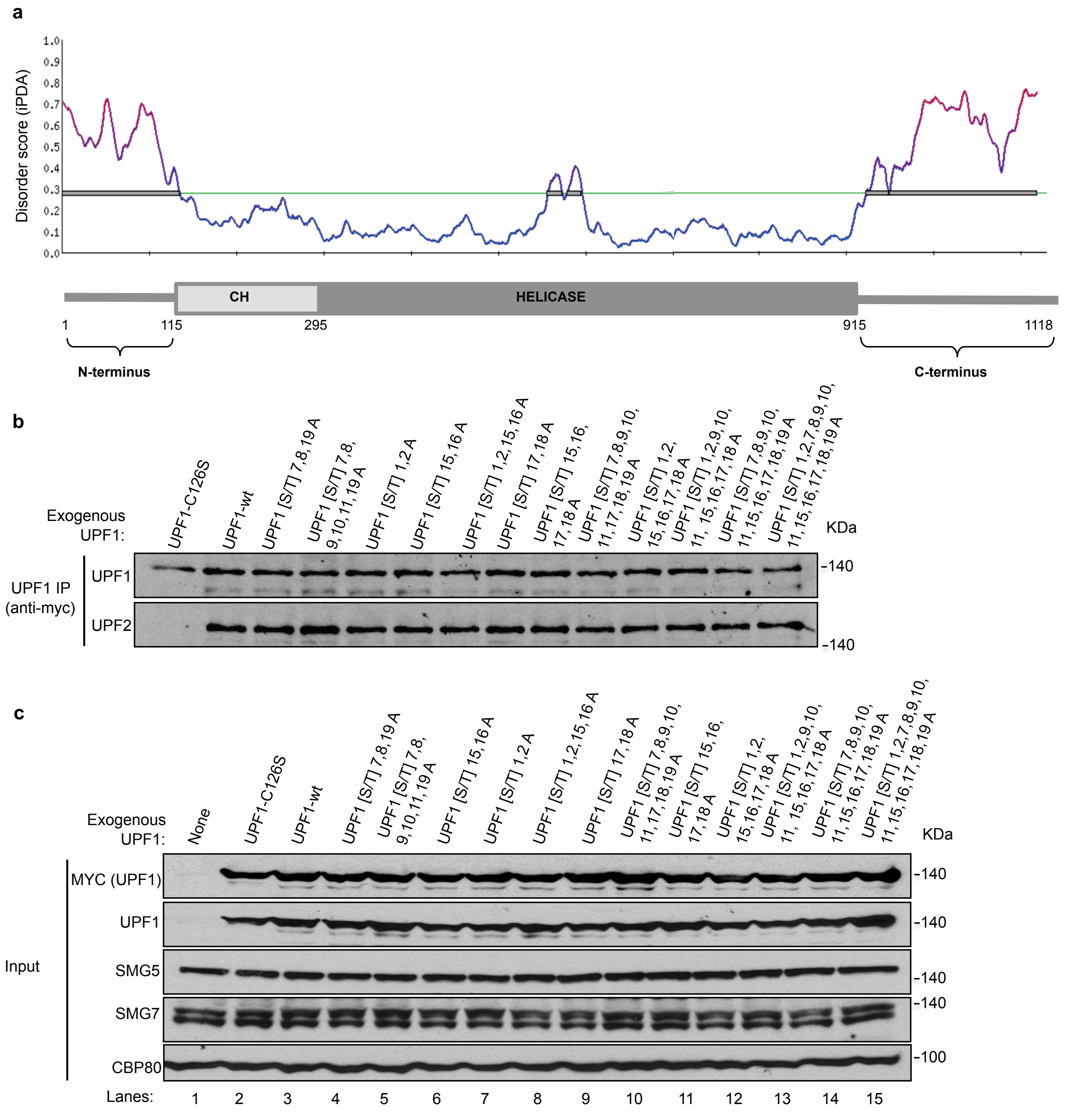

Supplementary Figure 4

(**a**) Plot showing protein disorder prediction of UPF1 analyzed with the iPDA web server (http://biominer.bime.ntu.edu.tw/ipda/) [1]. Cutting threshold (0.28) indicated in green is adjusted by integrating multiple secondary structure prediction programs (Jnet, PSIPred). Corresponding UPF1 domains are indicated by a schematic below the plot; CH, Cystein/Histidine-rich domain. (**b**) and (**c**) Western blots showing protein levels in input and myc-immunoprecipitated fractions from HeLa tet-off cells transfected with plasmids coding for the indicated UPF1 mutants.

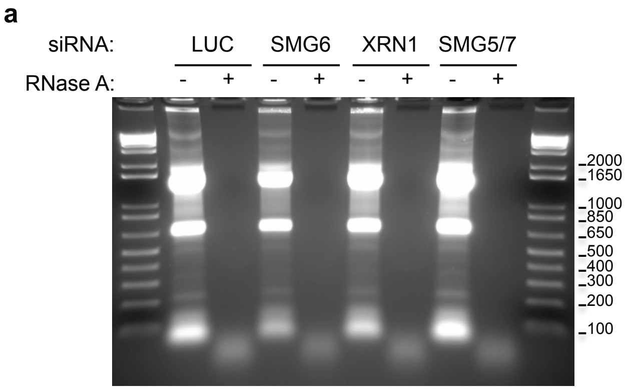

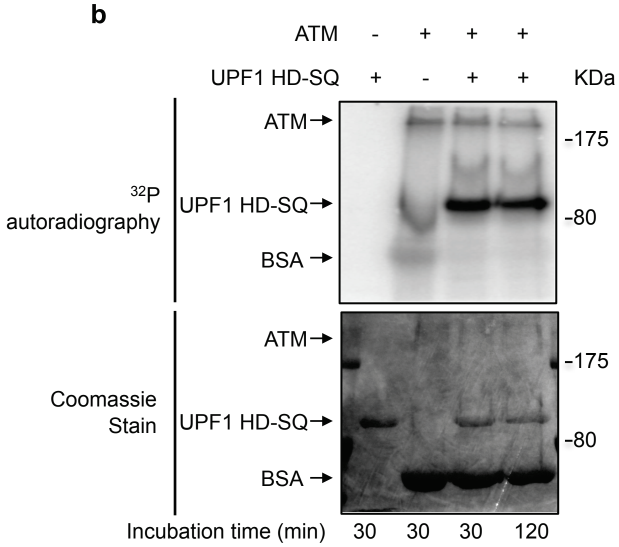

Supplementary Figure 5

(**a**) Ethidium bromide stained agarose gel showing efficiency of RNase A treatment in the co-IP assays shown in Fig. 5a.

(**b**) ^{32}P-autoradiograph showing phosphorylation of recombinant UPF1 (HD-SQ) by ATM *in vitro* and used in pull-down assays in Fig. 5d. Lower panel, coomassie stained gel.

Supplementary Figure 6

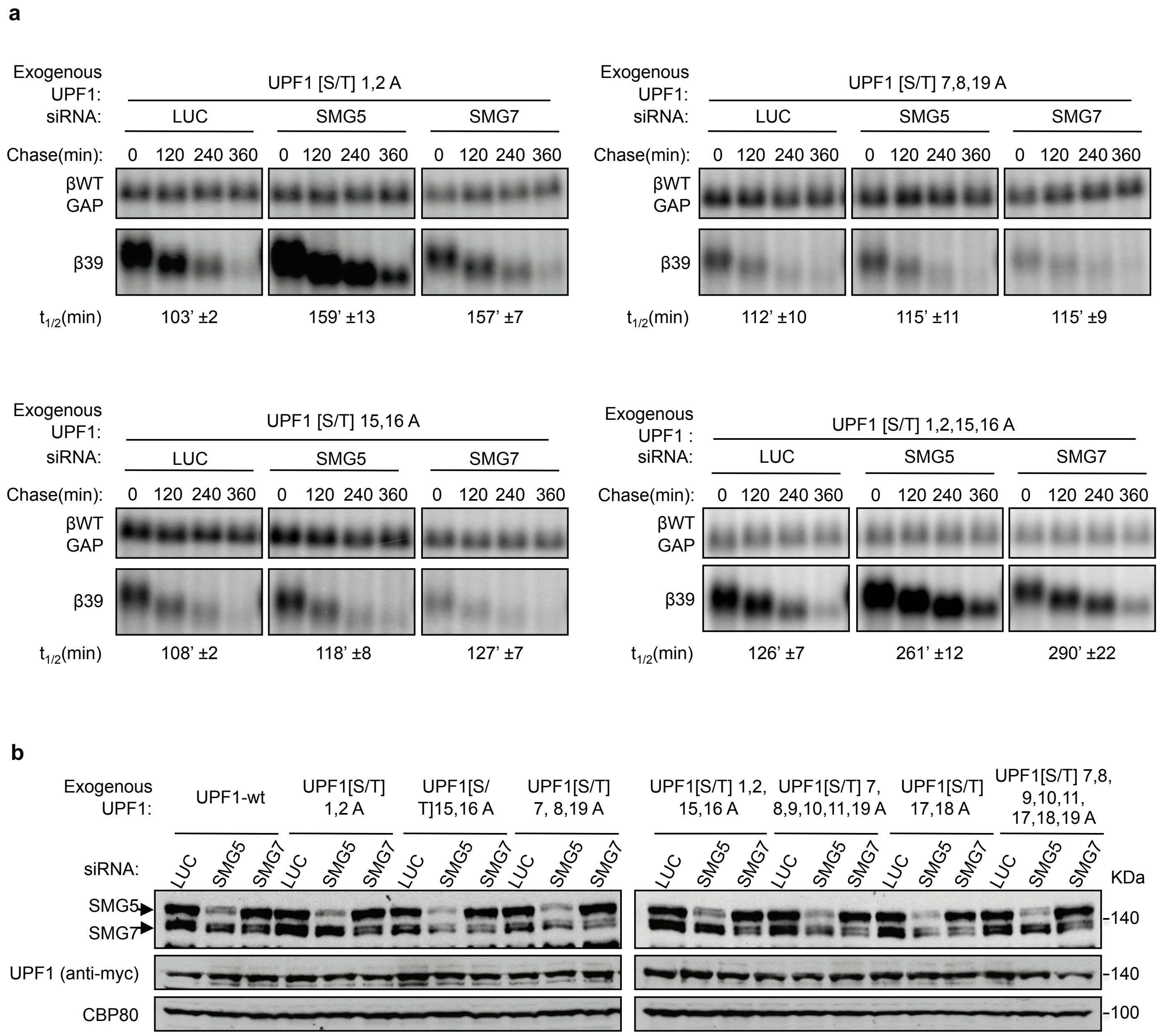

Supplementary Figure 6

(**a**) Northern blots showing the decay of β39 mRNA in HeLa Tet-Off cells depleted for endogenous UPF1 and expressing the indicated variants of exogenous UPF1. Cells were additionally transfected with siRNAs targeting Firefly Luciferase (LUC), SMG5 or SMG7. Numbers above panels refer to minutes after tetracycline-mediated transcriptional shutoff of β39 mRNA (chase). Values for $t_{1/2}$ were calculated after normalization of levels of β39 mRNA to levels of constitutively transcribed β-globin–GAPDH fusion mRNA (βWT-GAP) and are given ± S.E.M. from three independent experiments. (**b**) Western blots showing protein levels in HeLa tet-off cells transfected with plasmids expressing UPF1 variants used in Fig 6a and panel *a*.

Supplementary Figure 7

Uncropped scans related to Figure 1

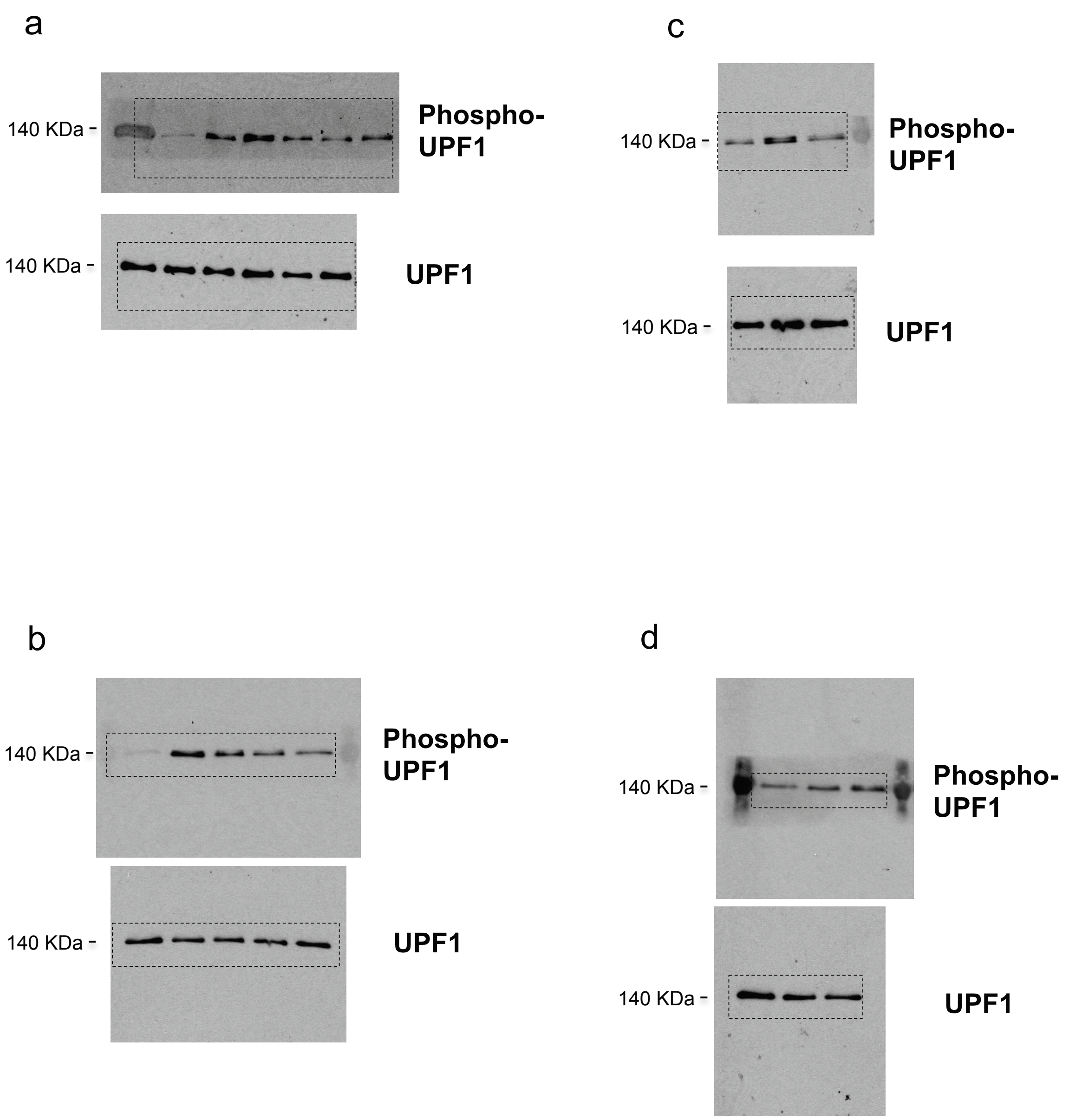

Supplementary Figure 7, continued

Uncropped scans related to Figure 2

a

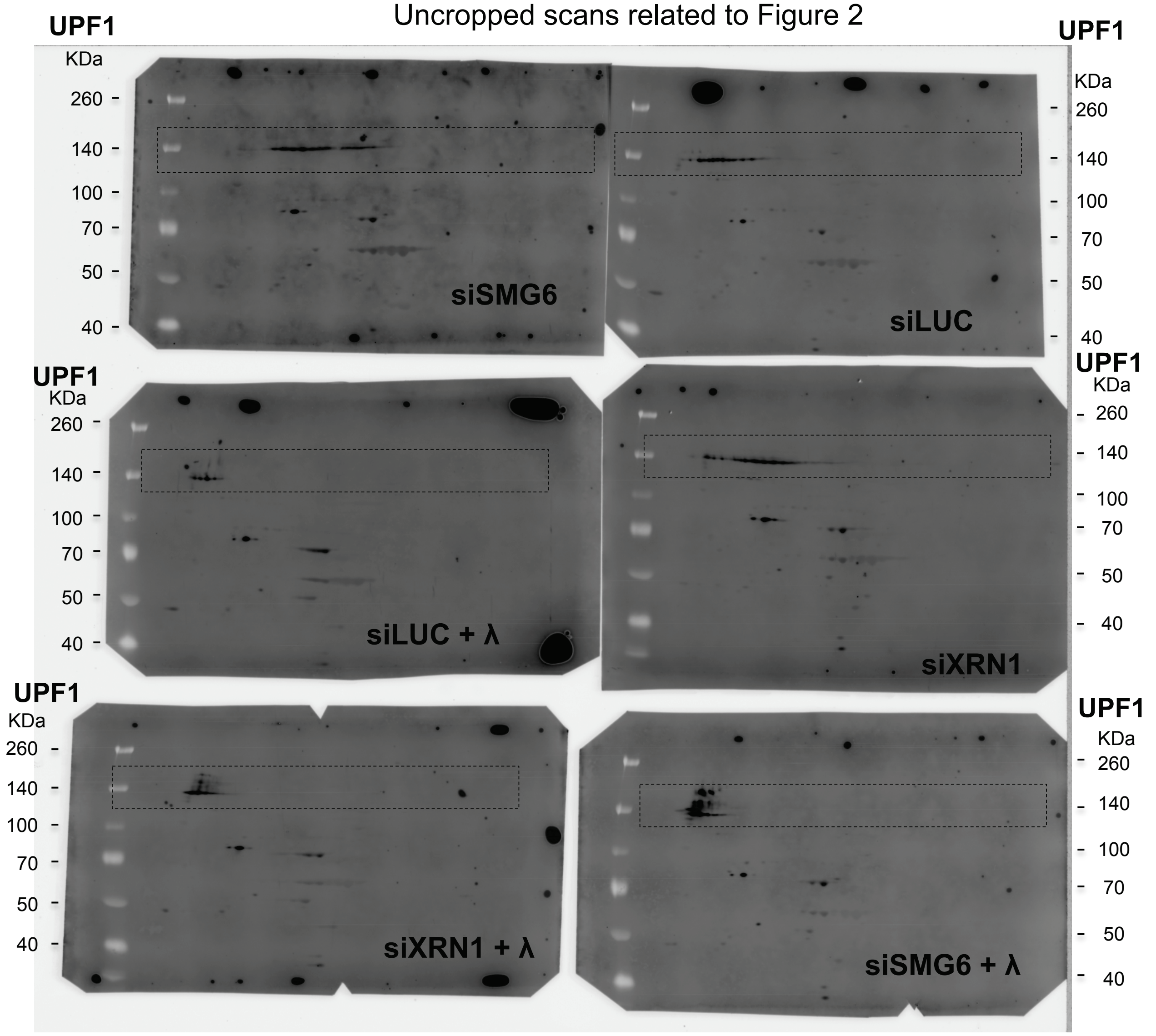

b

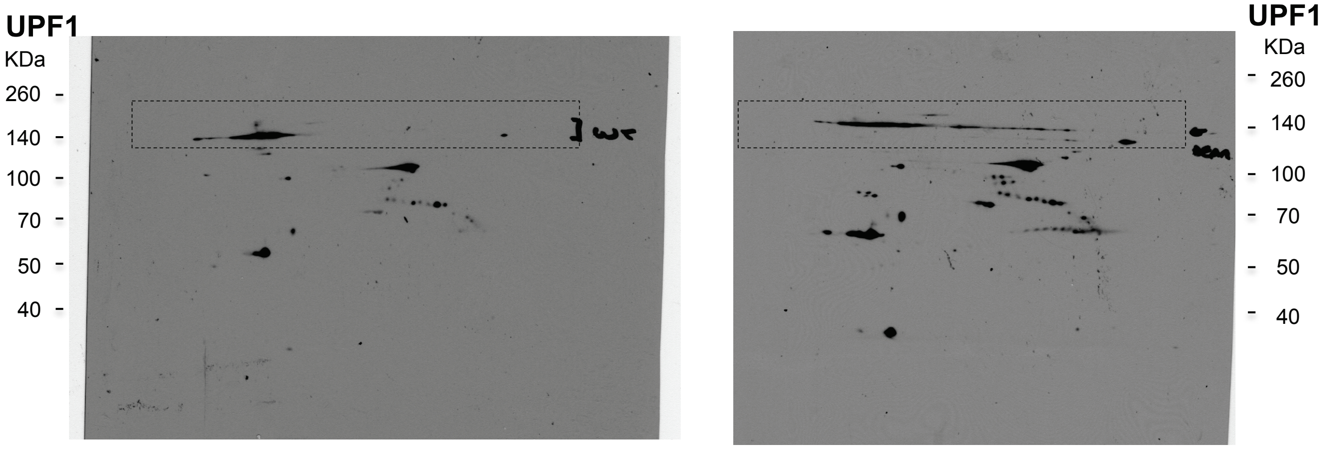

Uncropped scans related to Figure 3

a

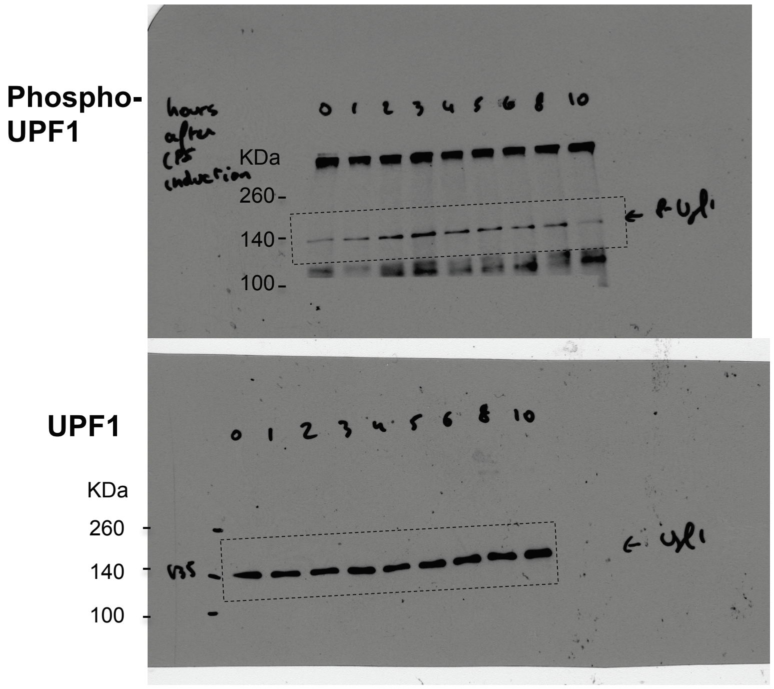

b

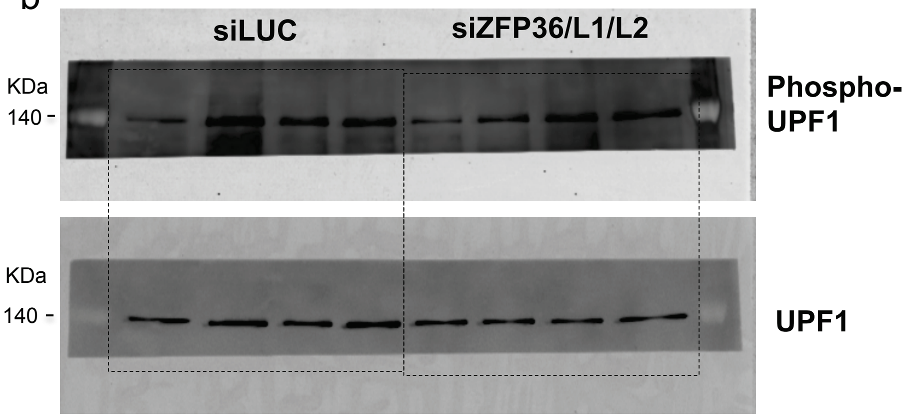

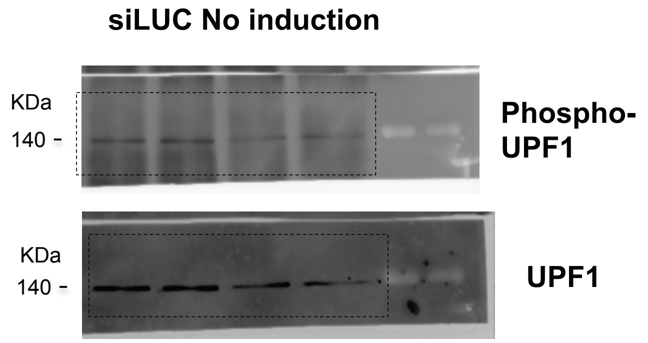

Supplementary Figure 7, continued

Uncropped scans related to Figure 4

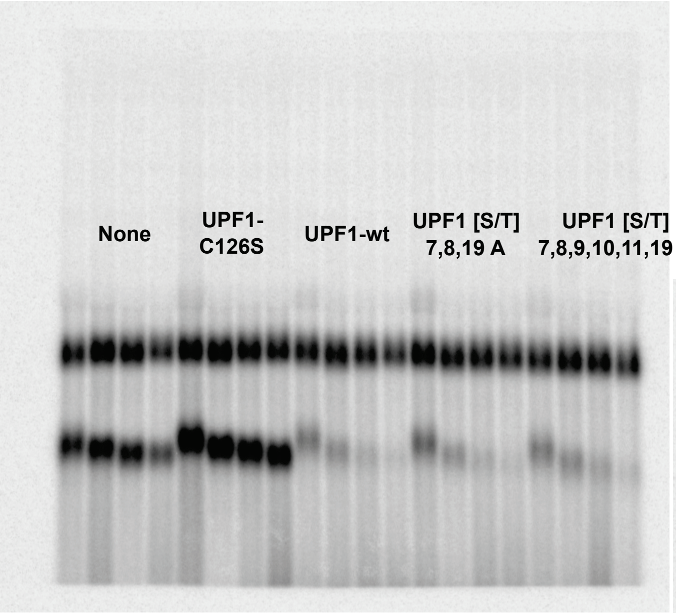

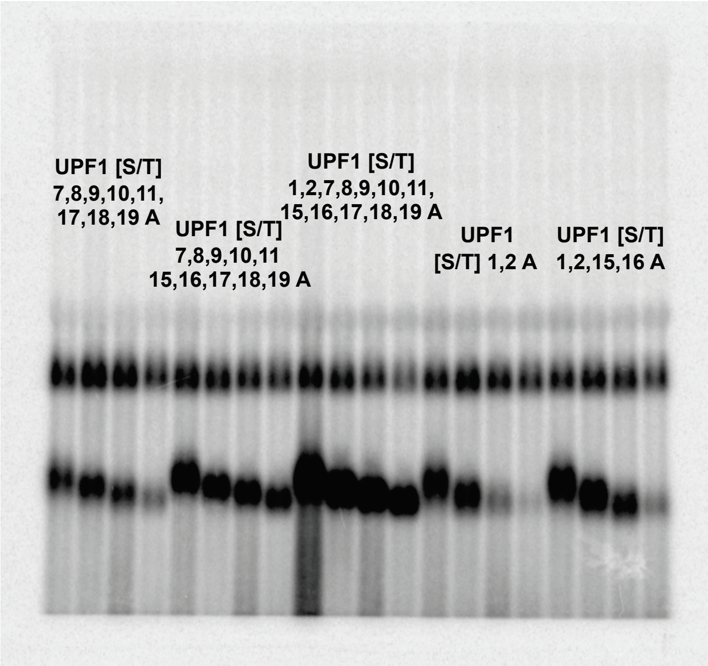

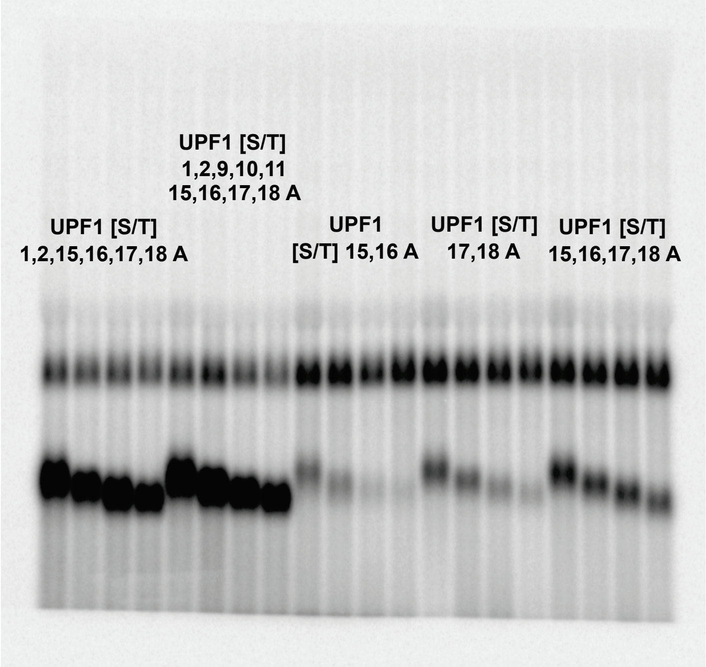

Supplementary Figure 7, continued

Uncropped scans related to Figure 5a

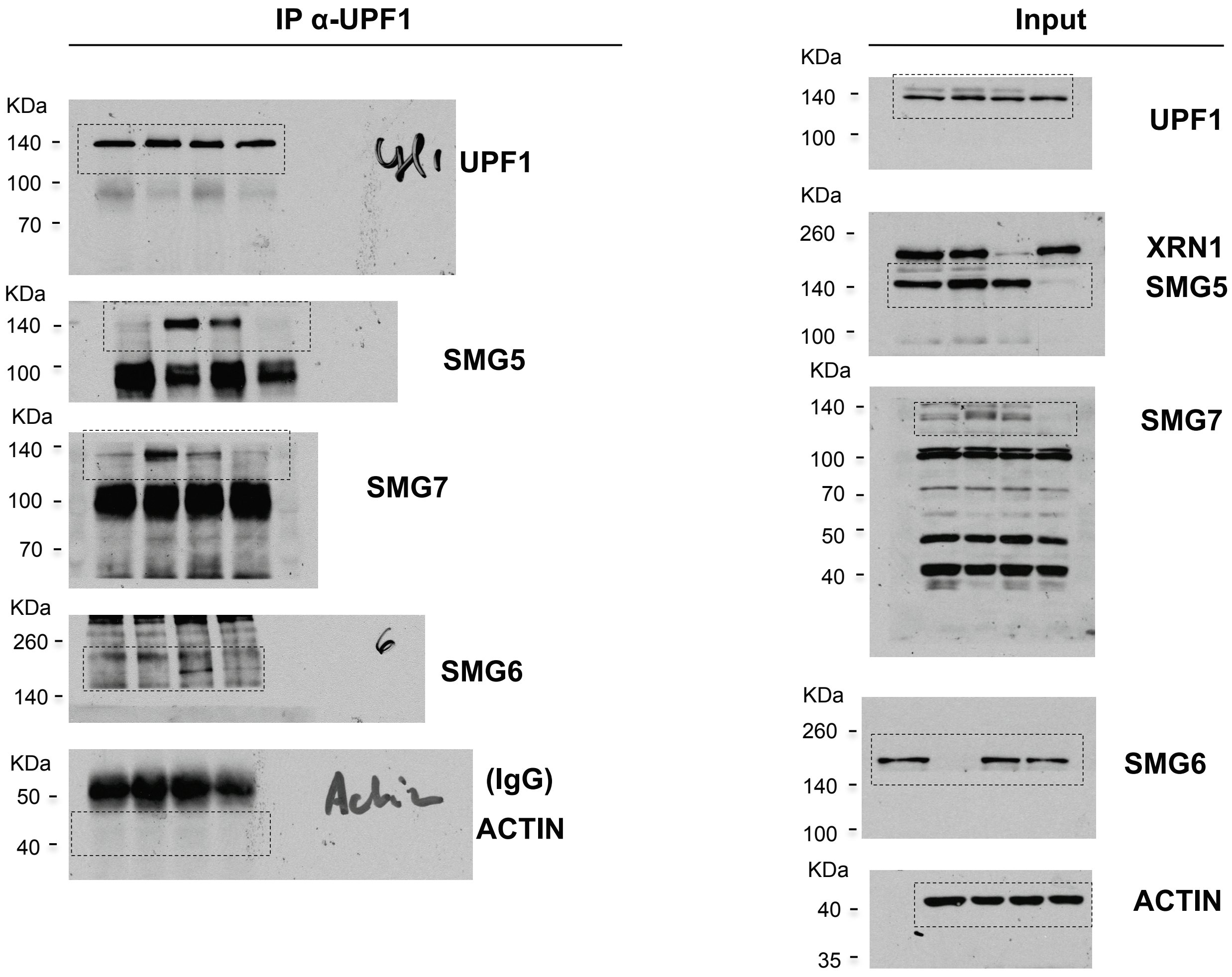

Supplementary Figure 7, continued

Uncropped scans related to Figure 5b

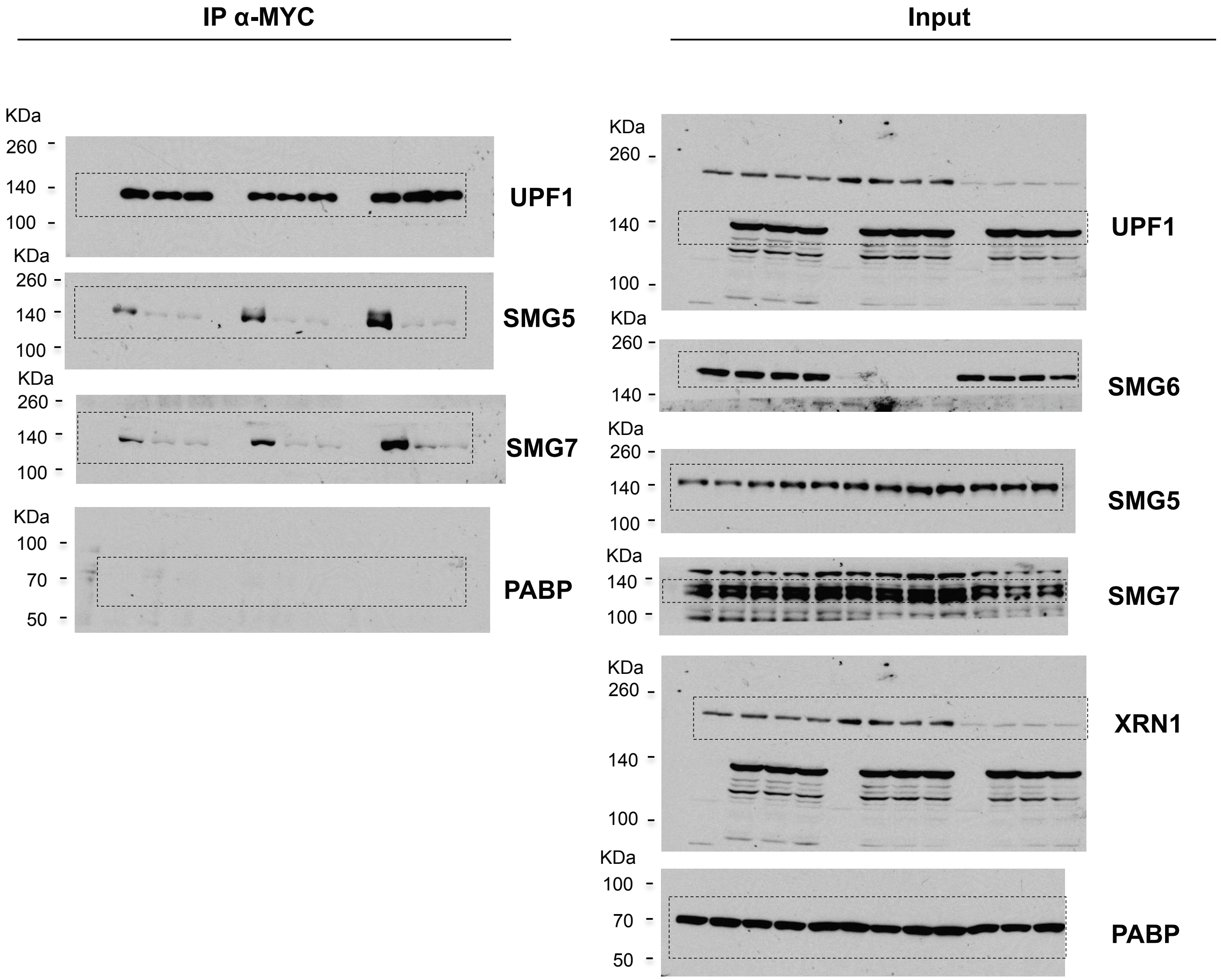

Uncropped scans related to Figure 5c

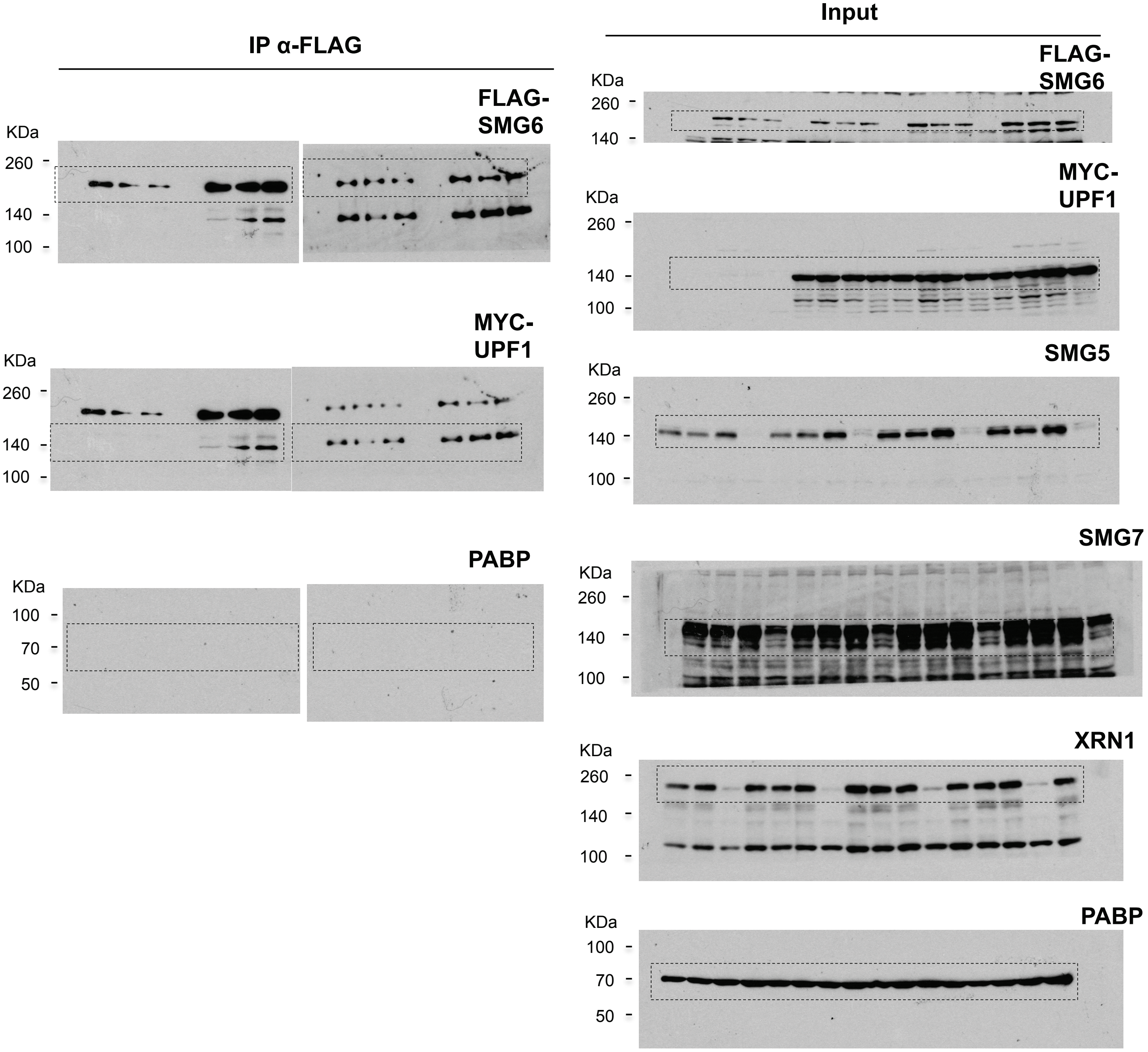

Supplementary Figure 7, continued

Uncropped scans related to Figure 5d

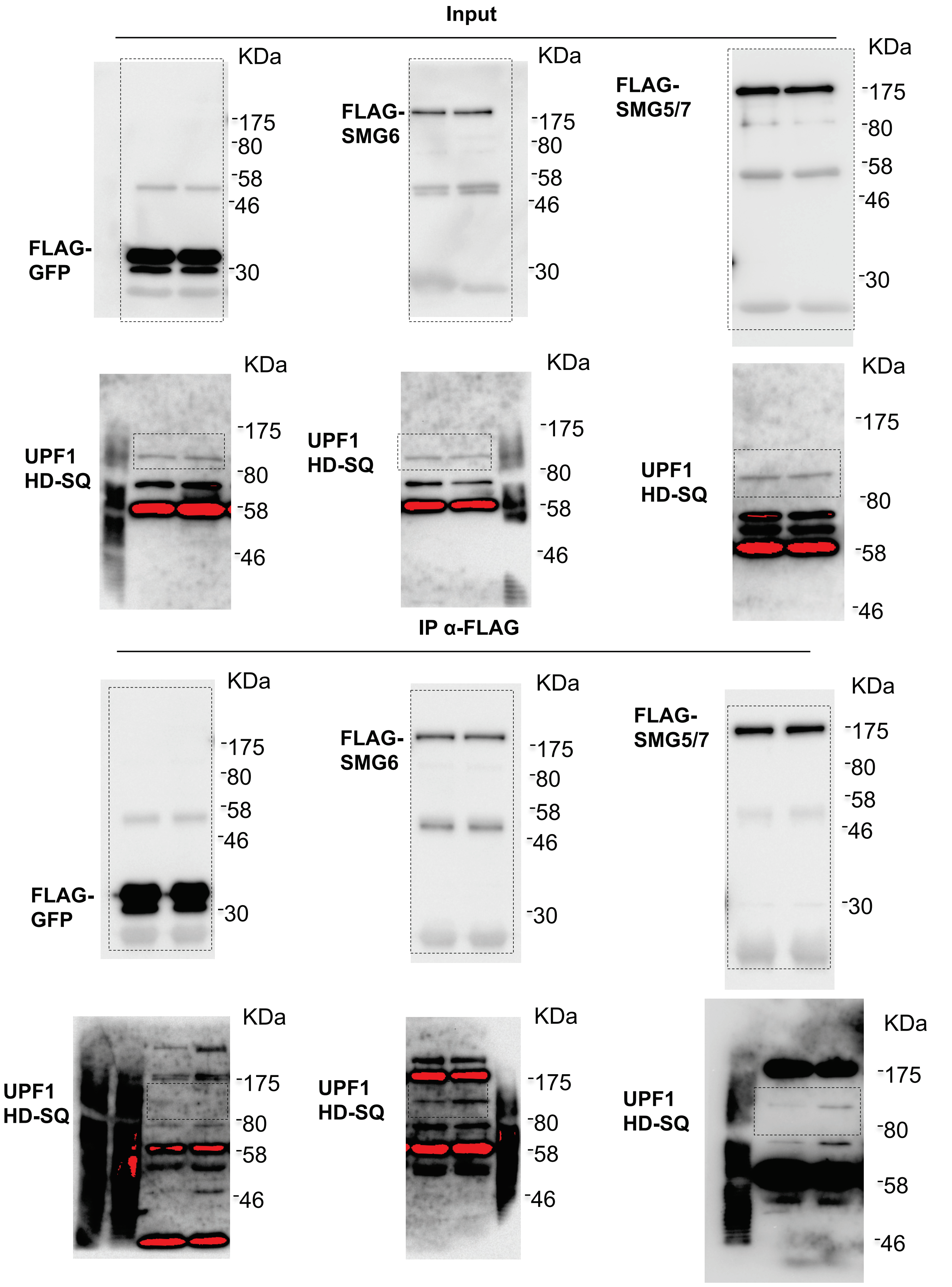

Supplementary Figure 7, continued

Uncropped scans related to Figure 6
(and Supplementary Figure 6)

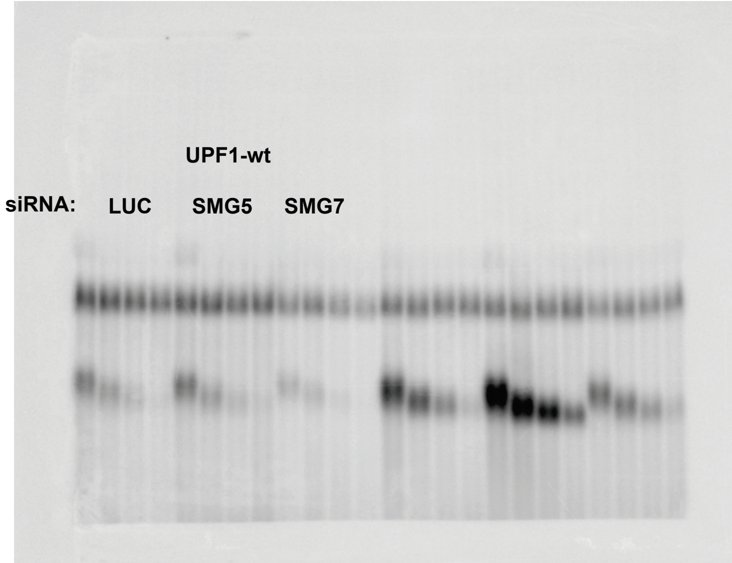

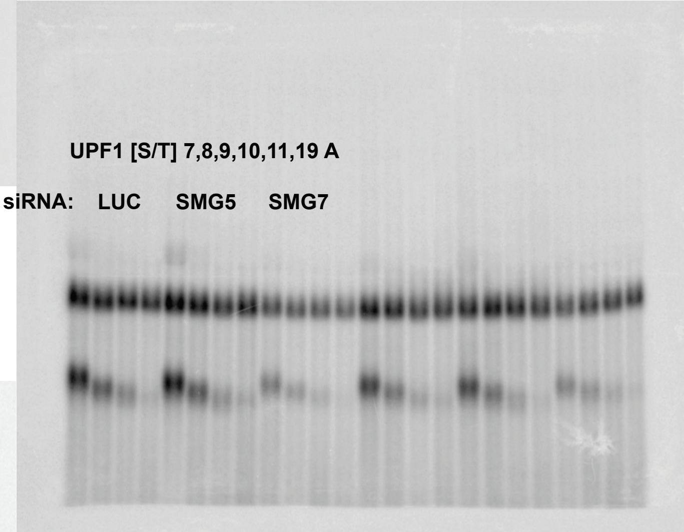

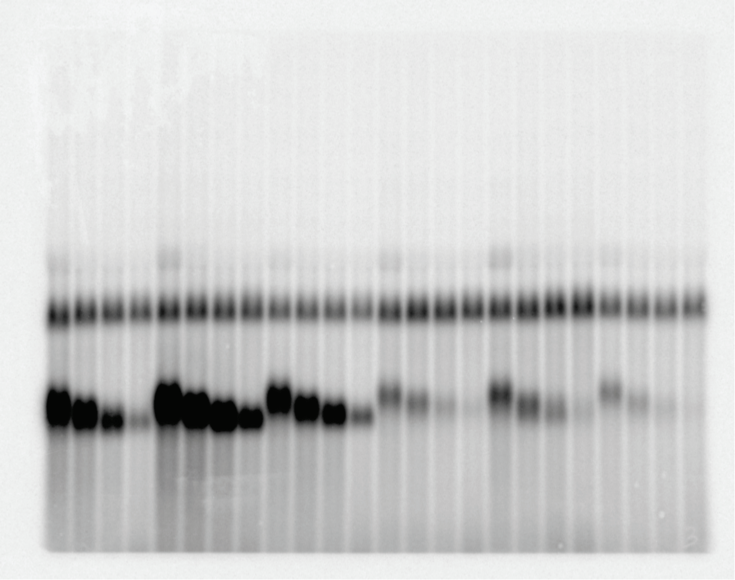

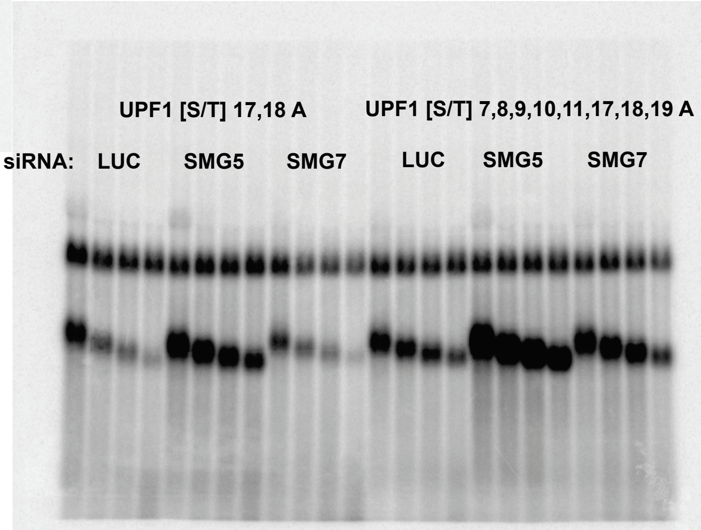

Supplementary Figure 7.

Uncropped scans related to Figures 1-6 as indicated.

Supplementary Reference

1. Su CT, Chen CY, Hsu CM. iPDA: integrated protein disorder analyser. *Nucleic Acids Res* **35,** W465-472 (2007).
